# The development of social attention in orangutans: Comparing peering behavior in wild and zoo-housed individuals

**DOI:** 10.1016/j.isci.2024.111542

**Published:** 2024-12-06

**Authors:** Paulina Kukofka, Richard Young, Julia A. Kunz, Lara Nellissen, Shauhin E. Alavi, Tri Rahmaeti, Fitriah Basalamah, Daniel B.M. Haun, Caroline Schuppli

**Affiliations:** 1 Department of Biology, University of Konstanz, Universitätsstraße 10 78464 Konstanz, Germany; 2 Development and Evolution of Cognition Research Group, Max Planck Institute of Animal Behavior, Bücklestraße 5 78467 Konstanz, Germany; 3 Department of Evolutionary Anthropology, University of Zurich, Winterthurerstraße 190 8057 Zurich, Switzerland; 4 Institute of Evolutionary Biology of Montpellier (ISEM), University of Montpellier, Campus Triolet 34095 Montpellier Cedex 5, France; 5 Department of Éco-Anthropologie et Ethnobiologie, Muséum National d’Histoire Naturelle, 57 rue Cuvier, 75005 Paris, France; 6 Institute of Biology, Department of Comparative Cognition, University of Neuchâtel, Rue Emile-Argand 11 2000 Neuchatel, Switzerland; 7 Department for the Ecology of Animal Societies, Max Planck Institute of Animal Behavior, Bücklestraße 5 78467 Konstanz, Germany; 8 Fakultas Biologi, Universitas Nasional, Jalan Sawo Manila, RT.14/RW.3, Jakarta 12550, Indonesia; 9 Department of Comparative Cultural Psychology, Max Planck Institute for Evolutionary Anthropology, Deutscher Platz 6 04103 Leipzig, Germany

**Keywords:** Wildlife behavior, Zoo animal behavior, Evolutionary biology

## Abstract

Social learning plays an essential role in all cultural processes, but the factors underlying its evolution remain poorly understood. To understand how socio-ecological conditions affect social learning, we compared peering behavior (i.e., close-range observation of conspecifics’ activities) in wild and zoo-housed Sumatran orangutans. Using long-term data describing over 3,000 peering events (performed by 65 individuals across settings), we found similar age trajectories of peering in both settings. Moreover, immatures universally preferred to peer at older individuals and in learning-intense contexts. However, zoo-housed immatures peered more frequently, and more at non-mother individuals than their wild conspecifics, even when social opportunities were controlled for. Therefore, although similarities across settings suggest that the tendency to attend to social information has hard-wired components, the differences indicate that it is also influenced by social opportunities and the necessity to learn. Our comparative approach thus provides evidence that socio-ecological factors and genetic predispositions underlie the dynamics and evolution of culture.

## Highlights

• Zoo-housed and wild immature orangutans peer in contexts where learning is expected
• Zoo-housed and wild immature orangutans mostly peer at older individuals
• Peering develops similarly over age but zoo orangutans peer more often than wild ones
• Social opportunities, the need to learn, and hard-wired tendencies affect peering

## Graphical abstract

**Figure:**
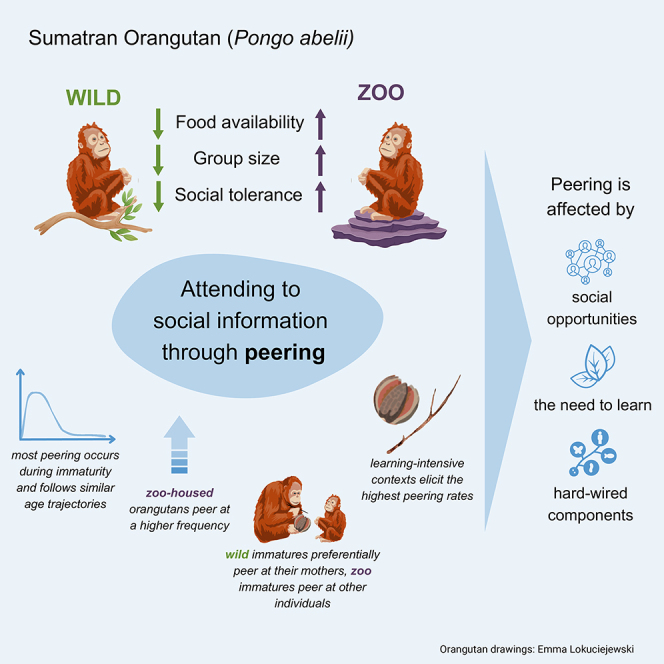

## Introduction

Like humans, many animals are born with an incomplete skill set and need to acquire a substantial share of the knowledge and behaviors necessary for survival and reproduction during their development.^1^^,^^2^ For this purpose, many species rely on social learning, which is “learning that is influenced by observation of, or interaction with a conspecific or its products.”^3^^,^^4^ Compared to independent learning, social learning is considered more efficient, as individuals can use social cues to guide their exploration and tap into existing skill sets within their population, allowing them to avoid the costs and potential risks of innovation and acquire skills faster.^5^^,^^6^^,^^7^^,^^8^^,^^9^ In its simplest form, social learning is non-observational, with knowledge transferred through facilitation or enhancement.^10^^,^^11^ However, evidence suggests that more learning-intense skills, such as multi-step tool use, are acquired through forms of social learning that likely allow for more detailed and reliable transmission of information, such as learning through observation or interaction, henceforth called “high-fidelity” social learning.^6^^,^^12^^,^^13^^,^^14^

Social learning is a prerequisite for the formation of cultures^4^^,^^15^^,^^16^^,^^17^ which are most pronounced and advanced in great apes, most of all humans.^15^^,^^16^^,^^18^^,^^19^ Cultural processes based on high-fidelity forms of social learning have played a pivotal role in the evolution of human cognition.^20^^,^^21^ So far, most studies infer social learning in wild animals by looking at its results, such as the distribution of cultural elements across populations or the spread of behaviors through populations (e.g.,^15^^,^^16^^,^^22^^,^^23^). However, social learning happens at the individual, not the population level, and entails attending to information provided by other individuals (i.e., social information). Therefore, to understand the evolution of human culture and cognition, we need to understand the conditions that affect individuals’ tendencies to attend to social information. Ecological and social conditions can affect attendance to social information on the immediate mechanistic level (i.e., prevailing conditions that increase or decrease attention to social information, henceforth called “immediate level”) and the developmental ontogenetic level (i.e., factors and conditions experienced during development that affect an individual’s current tendency to attend to social information, henceforth called “developmental level”).^24^ However, to be subject to classic evolutionary processes, the tendency to attend to social information must, at least to some extent, be genetically coded. By comparing how individuals of the same species attend to social information^25^ in environments with different social and ecological conditions, we can investigate behavioral flexibility, identify the conditions that affect social learning, and infer heritable predispositions.^26^

Such comparative studies of animals of the same species can help to shed light on factors affecting a behavior on the developmental and immediate level as genetic predispositions are controlled for.^27^ Comparing captive to wild individuals can be especially insightful because in contrast to wild animals, captive animals live in conditions that are usually far removed from their naturally occurring habitats, and their behaviors can differ greatly from those observed in the wild.^28^^,^^29^^,^^30^^,^^31^^,^^32^ These behavioral differences have been attributed to factors such as excess energy and free time,^30^^,^^33^ or habituation to humans.^34^^,^^35^^,^^36^^,^^37^ Captive conditions can help elucidate the factors affecting the expression of an observed behavior, as they can confirm or challenge patterns observed under natural conditions, such as the effects of different levels of ecological resource availability or social tolerance on the behavior.^38^

Orangutans are well suited for the study of social learning because of their high tendency to attend to social information, which plays an important role during their skill acquisition.^14^^,^^18^^,^^39^^,^^40^^,^^41^^,^^42^ Wild orangutans have a 6- to 9-year long dependency period, which provides the immature with drawn-out time for learning. Before weaning, orangutans stay in constant close proximity to their mother who is their main interaction partner.^14^^,^^39^^,^^43^ Adult orangutans are semi-solitary and have fission-fusion social dynamics.^44^^,^^45^ Depending on species and population, independent immatures and adults spend between 40% and 90% of their time on their own, resulting in a mean group size of less than two individuals,^45^ which limits their opportunities to attend to social information.^46^ Female orangutans are philopatric and their home ranges show substantial overlap with those of maternally related females who are their preferred association partners.^44^^,^^47^ During the dependency period, immatures have ample opportunities to learn from their mothers but their learning opportunities from other individuals are limited to the mother’s association partners. The slow development and low level of social interactions facilitate the tracking and documenting of learning opportunities, which is more challenging in species with a faster developmental period and higher association tendencies.

**Figure 1:**
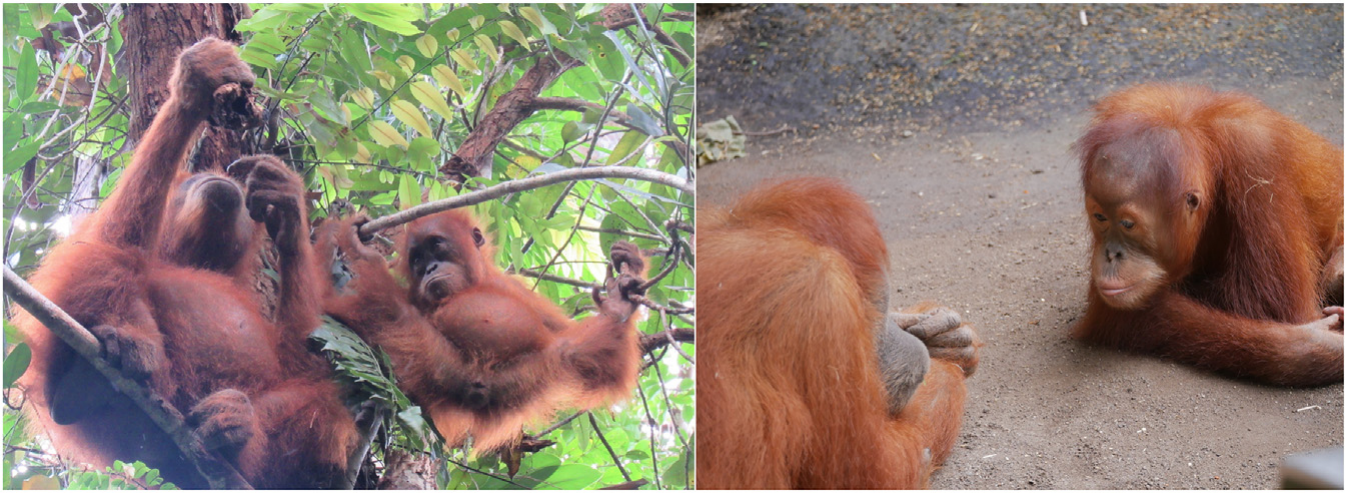
Peering in orangutans Immature orangutans peering in the feeding context in the wild (left) and in the zoo (right). Photos by Luz Carvajal (left), and Ivan Lenzi (right).

Observational learning through peering, i.e., close-range and sustained observation of a conspecific’s activities,^14^^,^^48^ is the most prominent and best-studied form of social learning that wild orangutans use (Figure 1). Peering has also been described in other primate species, including chimpanzees,^49^^,^^50^ bonobos,^51^^,^^52^^,^^53^^,^^54^ brown capuchin monkeys,^55^ white-faced capuchin monkeys,^56^ and vervet monkeys.^57^ On the proximate mechanistic level, peering is likely regulated by a mechanism other than a conscious desire to learn, and not all peering may lead to social learning. Furthermore, peering may have additional functions, including social functions.^54^^,^^58^ However, during peering, individuals attend to social information via sustained, close-range observation, which is a likely prerequisite for observational social learning. There is strong evidence that overall, peering is indeed a means for social learning: peering mainly occurs in contexts where learning is expected, such as foraging, nest-building, or social interactions,^14^^,^^19^ and its frequency increases with increasing processing intensity and rarity of a displayed behavior.^14^^,^^55^^,^^56^ Furthermore, peering rates are highest during immaturity, when individuals have to learn most of their skills,^14^^,^^41^^,^^49^^,^^50^ and peering is often followed by selective practice of the observed behavior.^14^^,^^41^ Given that peering is an indicator of social learning, investigating the factors that prompt an individual to engage in peering will therefore increase our understanding of the factors that modulate observational social learning.

Wild orangutans peer up to 40,000 times during their lifetime^25^ and show biases in their peering target selection (i.e., the individuals they peer at).^14^^,^^41^ With increasing age, peering preferences shift from the mother to non-mother individuals,^14^ with most non-mother peering first being directed at adults and later at peers,^41^ and with females and males having different peering target preferences.^59^ Comparative studies have found that the more gregarious and socially tolerant Sumatran orangutans (*Pongo abelii*)^45^ show higher peering rates than the less gregarious Bornean orangutans (*Pongo pygmaeus*),^48^^,^^60^ even after controlling for differences in association time (and hence opportunities to peer) and prevailing food availability.^61^ This difference suggests that either growing up in more sociable and ecologically favorable conditions increases individuals’ tendency to peer, or that the two orangutan species differ in their intrinsic tendency to peer.^60^^,^^62^ However, despite these findings, many aspects of how social and ecological conditions shape peering behavior on the immediate and developmental levels remain unclear. Furthermore, because these studies compared two different populations and species, it remains unclear whether genetic differences cause the observed group-level variation.

In this study, we compared the peering behavior of wild and zoo-housed Sumatran orangutans to investigate the factors that affect its expression. Zoo-housed and wild orangutans differ in their ecological and social environment. Zoo orangutans are housed in stable social groups with larger group sizes, which—unlike for other great apes—stands in stark contrast to their natural social environment and makes them an especially suited species for this study. Furthermore, wild orangutans have to acquire a range of learning-intense ecological skills (such as feeding on food items that require multiple processing steps before ingestion or building nests that are composed of multiple elements—both of which take individuals multiple years to master^63^^,^^64^). In comparison, zoo-housed orangutans rely on less learning-intense ecological knowledge as they are provisioned and thus have easy-to-process food (i.e., food that can be directly ingested) and nest materials abundantly available^65^ (Figure S1). We used a detailed comparative longitudinal dataset to look at the frequency of peering behavior across ages, the development of peering targets across ages, and the behavioral contexts in which peering occurred. By quantifying and controlling for social factors such as the number of peering targets available,^66^ we aim to disentangle whether ecological or social factors (i.e., the necessity to learn ecological skills^67^ and social opportunities to do so^68^) affect peering frequencies. We predicted the following:

(1) Peering as a means for social learning:
  If zoo-housed orangutans peer to learn, they will do so selectively and more frequently in learning-intense contexts, such as when rare foods are eaten or when foods require more intense pre-ingestive processing, as observed in wild orangutans.
(2) Ontogeny of peering:
  (a) Where peering is used for learning (prediction 1), its frequency will be highest during early immaturity and will decline quickly as individuals acquire their knowledge. This decline should occur earlier in zoo-housed orangutans, as they have less to learn (see earlier text).
  (b) If the necessity to learn ecological skills brings about peering behavior, zoo-housed orangutans will peer less than their wild conspecifics, as they have fewer ecological skills to learn (see earlier text).
  (c) If peering behavior is shaped by social opportunities (i.e., the availability of peering targets), zoo-housed orangutans will peer more than wild orangutans, due to their constant and close association with conspecifics (see earlier text). Peering frequency over age will thus show less defined age-dependent patterns in zoo-housed compared to wild orangutans.
  (d) If increased sociality and tolerance experienced during development leads to a higher overall tendency to peer, peering frequencies will remain higher in zoo-housed orangutans even after controlling for immediate social opportunities.
(3) Development of peering target selection:
  (a) If peering target selection is shaped by the necessity to learn ecological-relevant skills from knowledgeable individuals, with increasing age, immatures of both settings will increasingly peer at individuals other than the mother, as those may possess additional knowledge.^14^ Furthermore, individuals of both settings will preferentially peer at older individuals. However, zoo-housed orangutans will show less pronounced age-dependent shifts in their peering target selection, as well as a less pronounced bias to peer at older individuals, as they likely exhibit little inter-individual variation in skill and knowledge levels due to their less learning-intense ecological environment (see earlier text).
  (b) If peering target selection is shaped by social opportunities, individuals of both settings will show no age-dependent peering target selection and no bias to peer at older individuals once we control for immediate social opportunities to peer.
(4) Peering context selection:
  (a) If peering context selection is shaped by the necessity to learn, zoo-housed orangutans will peer less frequently in the feeding and nest-building context compared to wild orangutans, as these behaviors tend to be less learning-intense in zoos (see earlier text). Zoo-housed orangutans will show higher peering rates in social contexts because they are housed in larger, socially tolerant groups, allowing for a broader range of social interactions with more individuals involved than in the wild.
  (b) If peering context selection is shaped by social opportunities to peer, wild and zoo-housed orangutans will show less pronounced context preferences, once we control for social opportunities to peer in the different contexts.

## Results

### Peering as a means for social learning in zoo-housed orangutans

**Figure 2:**
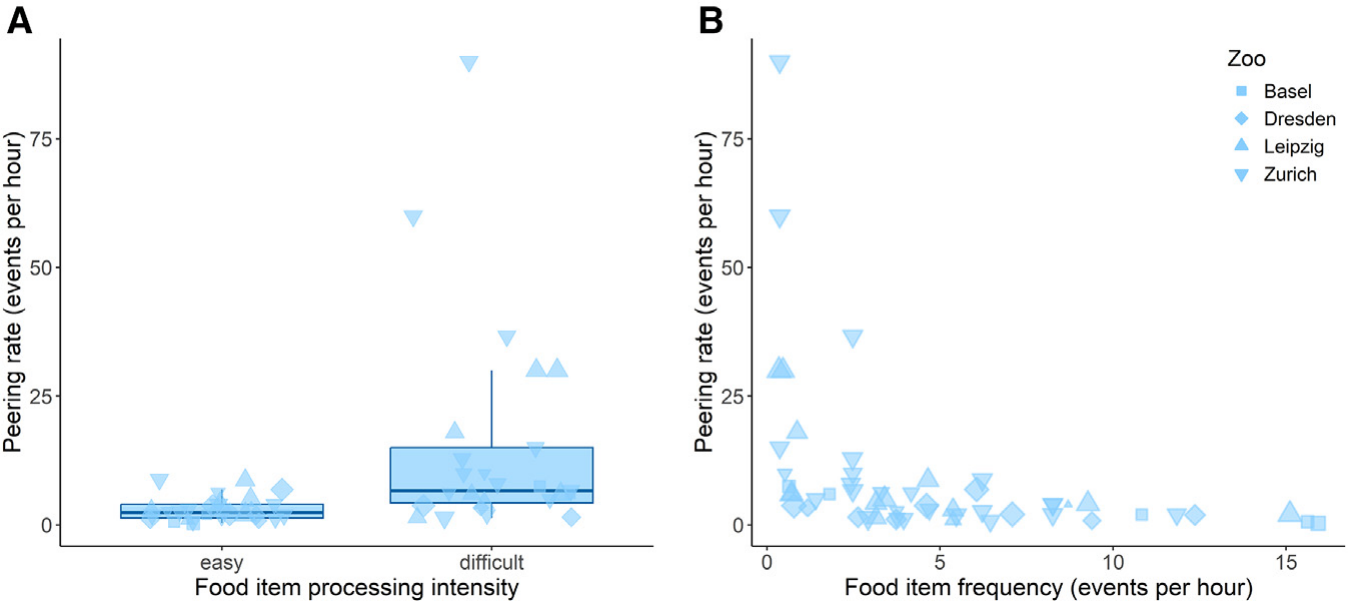
Peering rates in relation to food items’ processing intensity and frequency in association partners’ diet (a) Mean peering rates (events per hour the food item was consumed, with each data point representing one food item/individual combination) for easy and difficult-to-process food items of zoo-housed dependent immatures. The horizontal lines inside the boxes indicate the median, the boxes the interquartile range (IQR), and the whiskers extend to 1.5 times the IQR. (b) Hourly peering rates of zoo-housed dependent immatures over the frequency of food items in adult association partners’ diet. Symbol sizes correspond to the visible observed hours of each data point.

Our results revealed significantly higher peering at difficult-to-process food items compared to easy-to-process ones [GAMM: Estimate (difficult) = 0.502, *p* = 0.01; Table 1 and Figure 2A], as well as a significant decrease in peering with increasing daily frequency of the food item [GAMM: Estimate (Frequency) = −0.156, *p* < 0.001; Table 1 and Figure 2B] in immature zoo-housed orangutans, mirroring previous results on wild immature orangutans.^14^

**Table 1:** Peering as a measure for social learning: summary of model 1

Model	Response variable	Predictors	Type	Estimate	Standard error	*p* value
1 *n* = 54 r^2^_adj._ = 0.23 DE = 83.8%	Peering counts at food items (dependent immatures, zoo only)	Parametric terms: (Intercept) Processing intensity (difficult) Food item frequency Smooth terms: Z-Age Individual ID Observer ID	Intercept Fixed effect Fixed effect Fixed effect Random effect Random effect	1.544 0.502 −0.156	0.877 0.200 0.028	0.078 **0.012** **<0.001** **<0.001** **<0.001** 0.585

GAMM with processing intensity of food items (easy/difficult), frequency of food items in association partners’ diet, and age (z-transformed) as fixed effects and individual and observer ID as random effects. The time adult association partners spent feeding on easy or difficult food items was added as an offset term. Listed are estimates, standard errors, *p* values, sample size (n), adjusted r2, and deviance explained (DE). *p* values with significance at the 5% level are indicated in bold font.

### Ontogeny of peering

**Figure 3:**
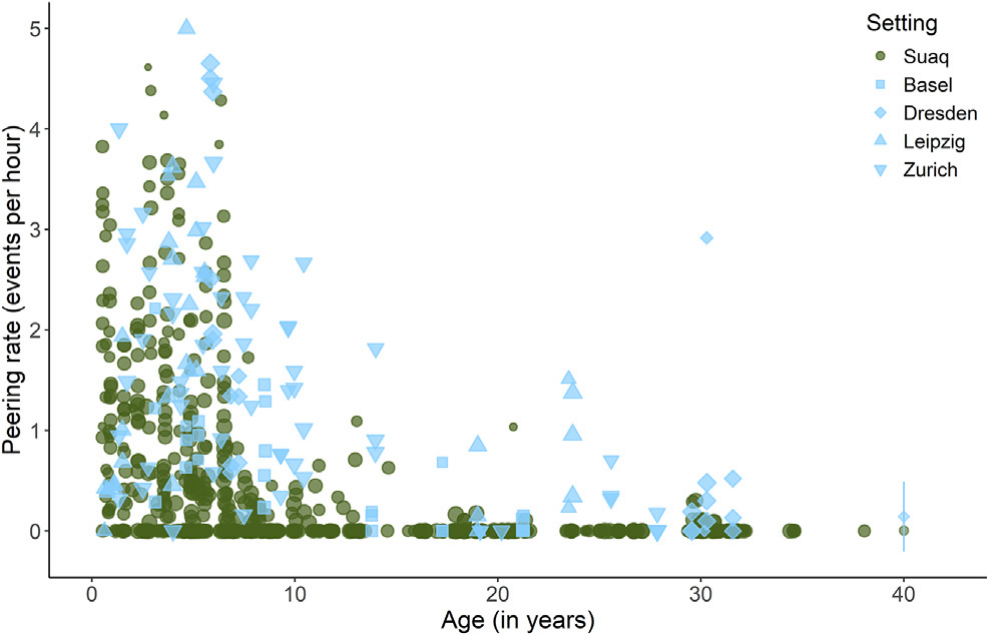
Development of peering frequency Peering rates (events per visible observation hour) over age in years by wild and zoo-housed orangutans. Each point represents one focal follow day. Mean data points for individuals older than 40 years with arrows representing the standard deviation. Symbol sizes correspond to the visible observed hours of each data point.

Peering frequencies developed similarly over the course of an individual’s life in wild and zoo-housed orangutans (Figures 3 and S2). However, zoo-housed individuals peered significantly more frequently than wild individuals [GAMM: Estimate (Zoo) = 2.395, *p* < 0.001; Table 2, Model 2a; Figure 3]. A comparison of the age splines for each setting showed significant differences until around the age of 6 years (Figure S3): Both in the wild and in the zoos, most peering occurred during the dependency period; however, peering rates in the wild peaked around the age of 3–4 years, whereas zoo-housed orangutans' peering rates peaked around the age of 5–6 years with a subsequent decrease of peering with age (Figure 3).

**Table 2:** Development of peering frequency over age: summary of models 2a and 2b

Model	Response variable	Predictors	Type	Estimate	Standard error	*p* value
2a *n* = 1061 r^2^_adj._ = 0.65 DE = 74.2%	Peering counts (all ages)	Parametric terms: (Intercept) Setting (zoo) Smooth terms: Z-Age Z-Age:Wild Z-Age:Zoo Individual ID Observer ID	Intercept Fixed effect Fixed effect Interaction Interaction Random effect Random effect	−3.346 2.395	0.254 0.389	**<0.001** **<0.001** **<0.001** 0.616 **0.006** **<0.001** **<0.001**
2b *n* = 538 r^2^_adj._ = 0.50 DE = 56.2%	Peering counts in peering range (immatures)	Parametric terms: (Intercept) Setting (zoo) Smooth terms: Z-Age Z-Age:Wild Z-Age:Zoo Individual ID Observer ID	Intercept Fixed effect Fixed effect Interaction Interaction Random effect Random effect	−0.790 1.252	0.256 0.277	**0.002** **<0.001** **0.001** 0.145 0.054 **0.017** **<0.001**

GAMMs with setting (Wild/Zoo), age (z-transformed), as well as an interaction between age and setting as fixed effects and individual and observer ID as random effects. Model 2a included the visible observation duration, and model 2b the time immatures spent in peering range as the offset term. Listed are estimates, standard errors, *p* values, sample size (n), adjusted r2, and deviance explained (DE). *p* values with significance at the 5% level are indicated in bold font.

**Figure 4:**
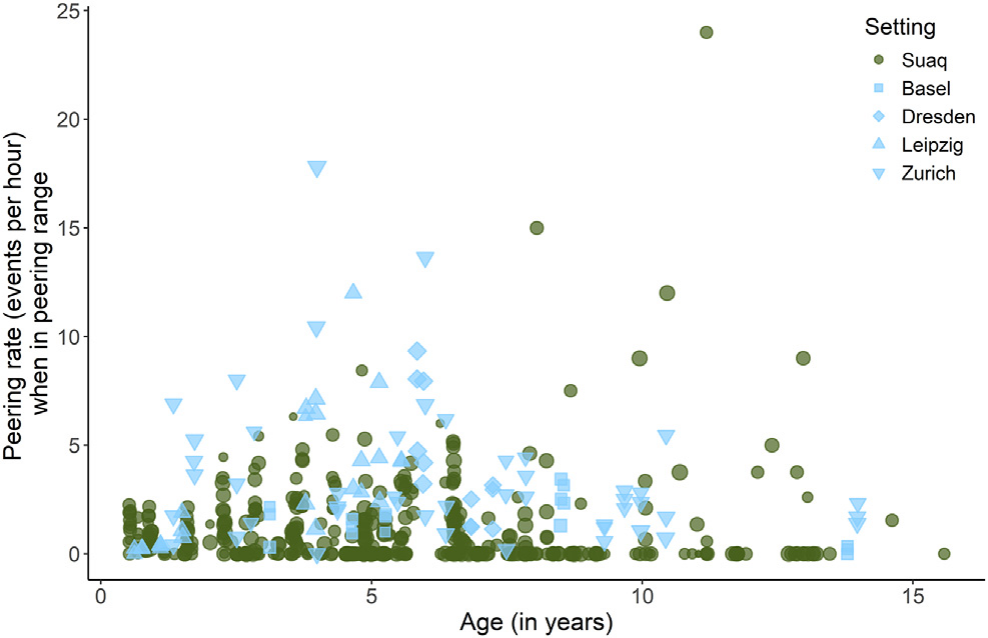
Development of peering frequency controlled for the availability of association partners over age Peering rates by wild and zoo-housed immature orangutans over age in years calculated as events per hour spent within peering range (0–2 m) of at least one other individual. Each point represents one focal follow day. Symbol sizes correspond to the visible observed hours of each data point.

When looking at peering controlled for the availability of association partners within peering range (see methods section “Development of peering target selection”), we found that zoo-housed immature orangutans peered significantly more frequently than wild immature orangutans [GAMM: Estimate (Zoo) = 1.252, p = < 0.001; Figure 4, Table 2, Model 2b]. However, both settings showed overall similar age trajectories of peering rates [Figure S4, GAMM: p (Age:Wild) = 0.144, p (Age:Zoo) = 0.054; Table 2, Model 2b].

### Peering target selection

**Figure 5:**
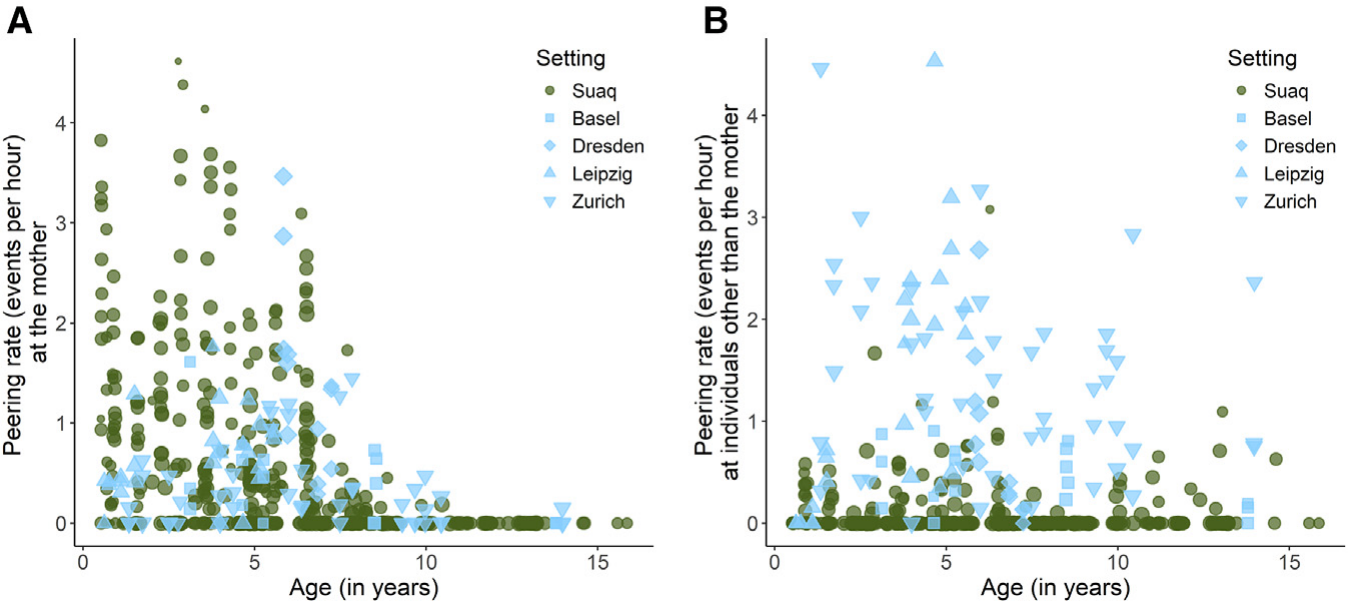
Development of peering frequencies at different peering targets over age Peering rates (events per visible observation hour) of immature wild and zoo-housed orangutans at (A) their mother and (B) individuals other than the mother across age in years. Each point represents one focal follow day. Symbol sizes correspond to the visible observed hours of each data point.

**Figure 6:**
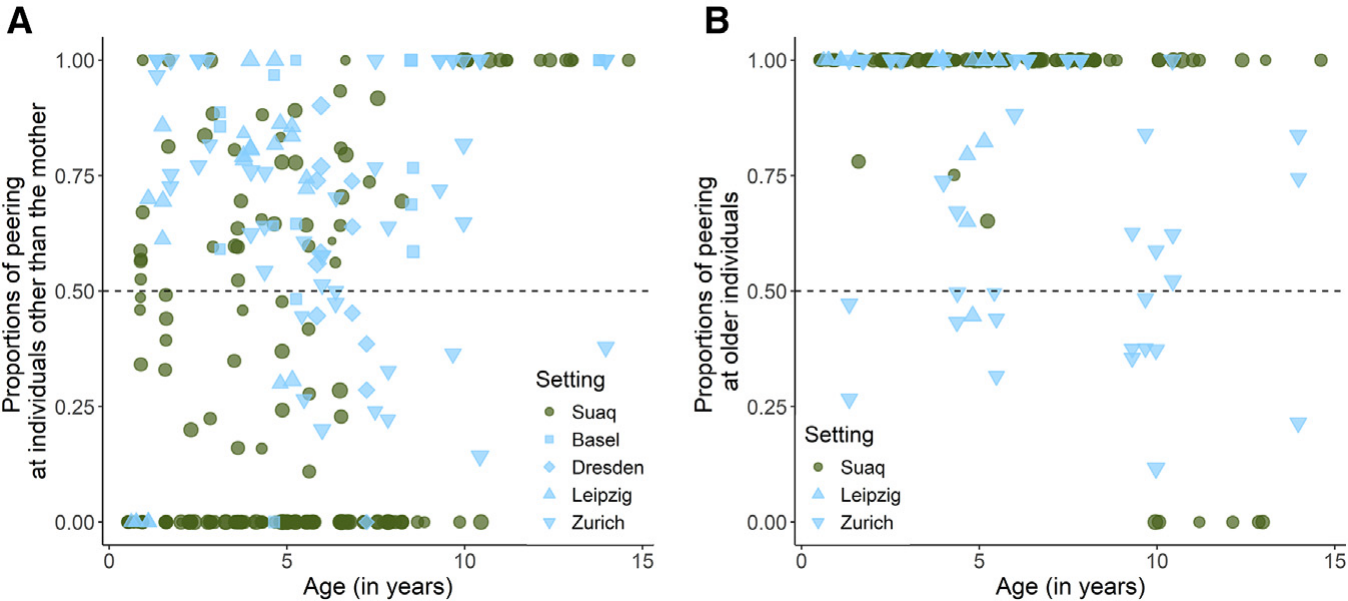
Development of peering frequencies at different peering targets over age controlled for opportunities to peer: (A) mother versus non-mother individuals and (B) older peering targets Proportions of peering by immatures directed at (A) non-mother individuals and (B) older individuals in the wild and in the zoo across age in years controlled for the time the peering individual spent in close proximity to each class of peering target. Each point represents one focal follow day. The size of the symbols reflects the total number of observed peering events for each data point. The dashed line at 0.5 indicates an equal proportion of peering at either peering target class.

When comparing peering rates directed at the immature’s mother (Figure 5A), we found that in both settings, peering decreased over time, but that the age trajectories differed: whereas in the wild, peering rates decreased linearly with increasing age, in the zoos, the effect of age on peering was non-linear [Figure S5, GAMM: p (Age:Wild) = 0.496, p (Age:Zoo) < 0.001; Table 3, Model 3a]. Peering rates directed at individuals other than the mother (Figure 5B) were significantly higher in immature zoo-housed orangutans compared to immature wild orangutans (GAMM, Estimate (Zoo) = 3.982, *p* < 0.001; Table 3, Model 3b), but neither setting showed a significant age effect (GAMM: p (Age) = 0.659; Table 3, Model 3b). To account for varying opportunities to peer at the two different target classes, we calculated peering frequencies controlled for the time an immature individual spent in peering range to the respective class of target (see methods section “Development of peering target selection”). We found that zoo-housed immature orangutans directed a significantly larger proportion of their peering at non-mother individuals compared to wild immature orangutans [GAMM: Estimate (Zoo) = 2.847, *p* < 0.001; Table 3, Model 3c, Figure 6A]. Furthermore, the age patterns differed between wild and zoo-housed immatures (Figure S6): in the wild, peering increased over time, whereas this clear shift was absent in the zoos (Figure 6A).

**Table 3:** Peering target selection: summary of models 3a–e

Model	Response variable	Predictors	Type	Estimate	Standard error	*p* value
3a *n* = 567 r^2^_adj._ = 0.60 DE = 66.8%	Peering counts at the mother (immatures)	Parametric terms: (Intercept) Setting (zoo) Smooth terms: Z-Age Z-Age:Wild Z-Age:Zoo Individual ID Observer ID	Intercept Fixed effect Fixed effect Interaction Interaction Random effect Random effect	−2.375 0.956	0.291 0.315	**<0.001** **0.002** **<0.001** 0.496 **<0.001** **<0.001** **<0.001**
3b *n* = 567 r^2^_adj._ = 0.28 DE = 65%	Peering counts at non-mother individuals (immatures)	Parametric terms: (Intercept) Z-Age Setting (zoo) Z-Age:Setting (zoo) Smooth terms: Individual ID Observer ID	Intercept Fixed effect Fixed effect Interaction Random effect Random effect	−3.951 −0.060 3.982 −0.111	0.307 0.136 0.412 0.252	**<0.001** 0.659 **<0.001** 0.660 **<0.001** **<0.001**
3c *n* = 335 r^2^_adj._ = 0.48 DE = 76.5%	Proportions of peering at non-mother individuals (immatures)	Parametric terms: (Intercept) Setting (zoo) Smooth terms: Z-Age Z-Age:Wild Z-Age:Zoo Individual ID Observer ID	Intercept Fixed effect Fixed effect Interaction Interaction Random effect Random effect	−2.021 2.847	0.251 0.359	**<0.001** **<0.001** **<0.001** **0.003** **<0.001** **<0.001** **<0.001**
3d *n* = 307 r^2^_adj._ = 0.58 DE = 64.6%	Proportions of peering at older individuals (immatures)	Parametric terms: (Intercept) Setting (zoo) Smooth terms: Z-Age Z-Age:Wild Z-Age:Zoo Individual ID Observer ID	Intercept Fixed effect Fixed effect Interaction Interaction Random effect Random effect	2.280 −0.868	0.291 0.511	**<0.001** 0.090 **<0.001** 0.084 1.000 **<0.001** **<0.001**

GAMMs with setting (Wild/Zoo), age (z-transformed), as well as an interaction between age and setting as fixed effects and individual and observer ID as random effects. Models 3a and b included the visible observation duration as offset term. Listed are estimates, standard errors, *p* values, sample size (n), adjusted r^2^, and deviance explained (DE). *p* values with significance at the 5% level are indicated in bold font.

When looking at peering directed at targets of different ages in relation to the peerer’s age (controlled for varying opportunities to peer at the different age classes), we found that both wild and zoo-housed immature orangutans directed most of their peering at older individuals (Figure 6B and Table 3) or at peers (Figure S7A and Table S1), but only a very low share of their peering at peers and younger individuals (Figure S7B and Table S1).

### Peering context selection

**Figure 7:**
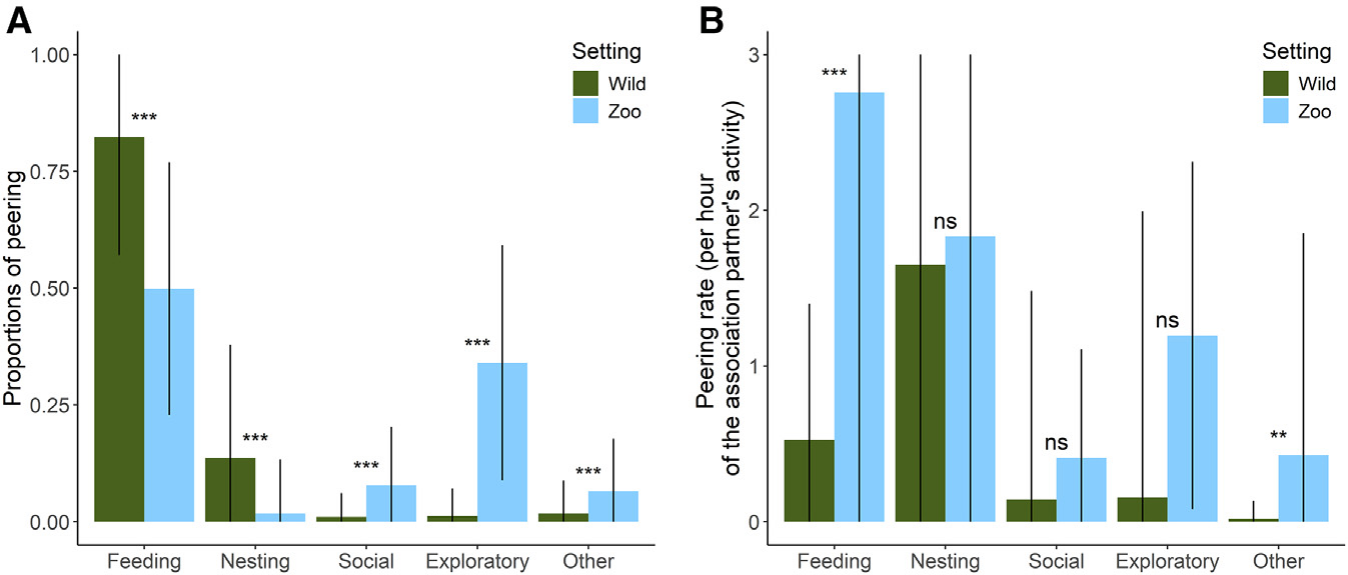
Contexts in which peering occurred Peering by wild and zoo-housed dependent immature orangutans in different contexts. (A) Overall proportions of peering and (B) rates of peering controlled for the time association partners spent engaging in the respective behavior. The bars represent the mean proportions (A) and rates (B) per focal follow day. Arrows represent the standard deviations; note that the top range of some standard deviations is cut off. Statistical significance is indicated by stars: ∗∗∗*p* ≤ 0.001, ∗∗*p* ≤ 0.01, ∗*p* ≤ 0.05, n.s. (not significant): *p* > 0.05.

We found that in both settings, most peering occurred in the feeding context (82.3% in the wild, 49.9% in the zoos). Wild immature orangutans directed about 13.7% of peering at nest-building behavior but hardly peered at social (1.0%) or exploratory behavior (1.2%). In contrast, zoo-housed immature orangutans frequently peered in exploratory (34.0%) or social (7.8%) contexts but only directed about 1.7% of peering at nesting behavior (Figure 7A). These differences in peering proportions were significant for all contexts (Figure 7A and Table S2, Models 4a–e). Note that the response variables in the five models were not independent of each other, however, the *p* values of the models remain significant even after correcting for multiple comparisons.

When looking at rates of peering controlled for the time association partners engaged in the behavior, wild and zoo-housed immatures did not differ significantly in the contexts they peered at (Figure 7B and Table S2, Models 4f–j), with the exception of the feeding context where zoo-housed immatures peered significantly more (GAMM: Estimate (Zoo) = 1.634, *p* < 0.001; Table S2, Model 4f). Both wild and zoo-housed immature orangutans peered mostly at feeding and nest-building behavior (Figure 7B).

## Discussion

In this study, we compared the peering behavior of wild and zoo-housed Sumatran orangutans to investigate what factors regulate individuals’ attendance to social information and to ultimately shed light on the factors affecting orangutan observational social learning. Specifically, we aimed to disentangle the extent to which peering is influenced by social and ecological immediate and/or developmental factors, as opposed to being hard-wired (i.e., not affected by immediate and/or previously experienced conditions^69^ and thus at least to some extent genetically determined).

### Peering as a means for social learning

Attending to social information via observation is a cornerstone of observational social learning, whereas the difference between the observed information and the existing knowledge of the peering individual, as well as the ability of the peering individual to act upon the observed information, determine whether learning takes place. Wild immature orangutans selectively peer in learning intense contexts, such as rare and difficult-to-process food items.^14^ As predicted, we found that zoo-housed dependent immatures peered more at difficult-to-process food items and that their peering frequency decreased with increasing familiarity of the food item (Figure 2). These findings support the assumption that both wild and zoo-housed immature orangutans use peering to learn.

### Ontogeny of peering

We found that peering rates in both wild and zoo-housed orangutans followed similar age trajectories, with most peering occurring during the dependency period (Figure 3). This finding suggests that the age-dependent relative tendency to peer is independent of immediate or developmental social or ecological factors and thus likely follows a hard-wired developmental trajectory. However, peering rates in wild orangutans peaked slightly earlier, around 3–4 years of age, compared to 5–6 years in their zoo-housed conspecifics. This is surprising, considering that zoo-housed orangutans tend to show shorter developmental periods compared to their conspecifics in the wild, which suggests an overall faster physical development.^70^^,^^71^^,^^72^ The later peak in peering in the zoo setting may be a consequence of increased social tolerance in zoo-housed individuals (see discussion section “Development of peering target selection” below).

Despite the similar relative age trajectories, overall peering rates were higher in zoo-housed orangutans (Figure 3), indicating that either immediate and/or developmental social factors influence the absolute frequency at which the behavior is expressed, rather than the necessity to learn ecological skills and knowledge (given that zoo-housed orangutans have to acquire less learning-intense ecological skills and knowledge). Schuppli et al.^60^ found that in the wild, immatures of the more gregarious and tolerant Sumatran orangutan species show higher peering rates compared to the less social Bornean orangutans, even when controlling for social opportunities.^60^ Although the 2017 study could not differentiate between developmental and hard-wired (i.e., species-level) effects on peering rates, the results of our study comparing the same species across different settings shed novel light on this distinction. Even after we controlled for differences in social opportunities, zoo-housed immature orangutans exhibited higher peering rates compared to wild immatures (Figure 4), suggesting that peering frequency is not solely a consequence of immediate social opportunities to peer.

Our results suggest that increased sociality, experienced both currently and during development (via increased social tolerance by group members other than the mother and particularly during late immaturity and adulthood—see also discussion section “Development of peering target selection” below), as well as more available free time,^73^ may influence peering frequencies. A recent study found that, in line with the developmental predictions of the Cultural Intelligence Hypothesis,^5^ zoo-housed Sumatran orangutans explore more frequently, show a greater variety of exploratory actions, and engage in more tool-oriented behaviors compared to their wild conspecifics (I.L., unpublished data). Furthermore, zoo-housed Sumatran orangutans outcompete zoo-housed Bornean orangutans in problem-solving tests,^74^ which suggests that differences in sociality ultimately lead to species-level differences in cognitive abilities (as predicted by the evolutionary version of the Cultural Intelligence Hypothesis^5^).

### Development of peering target selection

In both wild and zoo-housed immature orangutans, peering directed at the mother decreased with increasing age of the individual (Figure 5A), which implies a hard-wired predisposition to peer at the mother during early dependency. The higher peering rates at the mother by zoo-housed immatures were likely a consequence of their overall higher peering frequency (see earlier discussion section “Ontogeny of peering”). Peering requires that the peering target tolerates the peering individual in close spatial proximity. For wild immature orangutans, the mother is the main association partner, and thus her level of tolerance likely shapes peering opportunities. The mother’s tolerance for food interactions (which like peering requires close proximity) peaks during the time her offspring learns feeding skills, at around 3–5 years.^39^ This coincides with the peak in wild peering rates observed here (Figure 5A). After that, tolerance gradually decreases as the mother energetically prepares for a new pregnancy.^75^^,^^76^^,^^77^ Since they are provisioned, zoo-housed mothers may not experience the same energetic constraints and can remain highly tolerant throughout their offspring’s dependency period. Additionally, zoo-housed immature orangutans are in constant association with other individuals, so peering opportunities are not solely dependent on the mother’s tolerance. In the wild, interaction with the mother decreases with increasing group size, suggesting that social dynamics influence immature orangutan behavior.^78^

Peering rates at non-mother individuals were higher in zoo-housed immatures compared to wild immatures, and this difference was more pronounced than for peering at the mother (Figure 5B). Furthermore, peering at individuals other than the mother increased with age in the wild, whereas there was no discernible pattern in zoo-housed immatures, suggesting that social and/or ecological factors influence the more nuanced patterns of peering target selection across different ages. When controlling for social opportunities, immature zoo orangutans exhibited a higher preference to peer at non-mother individuals compared to wild immatures (Figure 6A). In the wild, peering at non-mother individuals gradually increases with age, and after the age of 10 years, individuals almost exclusively peer at them,^14^^,^^41^ coinciding with increased ranging independence from their mothers.^46^ However, this age-specific pattern was absent in zoo-housed orangutans. Wild orangutans show a strong tendency to peer at relatives,^41^ and tolerance toward kin is usually higher than toward unrelated individuals.^79^ Most of the orangutans from our zoo populations were either to some degree related or spent most of their lives together. Growing up closely surrounded by the same individuals may establish group members as trusted role models, which may in turn lead to higher peering rates. In the wild, favorable environmental conditions, such as food availability, positively affect social tolerance and social learning opportunities between association partners, particularly between unrelated ones.^61^ In contrast, zoo-housed orangutans are provisioned and experience little to no resource competition, which may significantly increase overall tolerance levels.

We found that immatures in both settings preferentially peered at older individuals and peers but rarely at younger conspecifics (Figures 6B and –S7). This is in line with previous findings on selective bias in chimpanzees^80^ and capuchin monkeys.^81^^,^^82^ Such a tendency is advantageous for species adapted to learning-intense foraging niches, because in natural settings, older individuals are likely to be experts and learning from them is more efficient and safer.^83^ However, compared to their wild conspecifics, zoo-housed orangutans have less variable food available, which usually requires little pre-ingestive processing (see Figure S1 for a comparison of the diet processing intensity of the two settings) and are provided with simpler nesting material.^64^^,^^65^ Consequently, orangutans growing up in zoos may possess complete skill sets at an earlier age compared to their wild conspecifics, making it less likely that peering individuals will indeed learn more by peering at older individuals compared to younger individuals or peers. The fact that we nevertheless find such a clear bias in both settings suggests a hard-wired tendency to attend to older individuals, which (under natural conditions) most likely provides individuals with fitness benefits. All in all, our results suggest that both the need to acquire ecological knowledge, as well as social opportunities i.e., the availability and tolerance of peering targets, affect peering target selection.

### Peering context selection

Wild and zoo-housed immature orangutans differed in the contexts in which they peered (Figure 7A). When looking at absolute peering proportions, in line with our prediction that the necessity to learn shapes peering context selection, immatures in the zoos spent less time peering in feeding and nest-building contexts compared to wild orangutans and more time peering at social and exploratory behaviors. Nest building in the wild is an intricate and multi-step process that includes bending and intertwining branches, as well as adding layers of pillows and blankets made from leafy twigs and loose leaves.^84^ In zoos, nest building mainly consists of gathering wood wool, blankets, or other soft materials provided for the animals.^65^ Similarly, feeding skills are less processing-intense in zoos compared to the wild (Figure S1). Zoo-housed orangutans likely need to peer less to reach adult-like levels of proficiency in these skills and, consequently, may acquire them earlier than their wild conspecifics, leaving them with more time to direct their attention to other behaviors. Similarly, wild Bornean orangutans, whose foraging techniques contain fewer steps of pre-ingestive processing compared to those of Sumatran orangutans, show lower proportions of peering in the feeding context.^14^ Exploratory and social behaviors are generally rare in wild orangutans,^48^^,^^85^ due to their strong neophobia and solitary lifestyle. In wild dependent immatures, opportunities to peer in social contexts are restricted to their mothers’ associating with conspecifics.^49^ Indeed, relative to the time association partners spent engaging in the respective behavior (i.e., controlled for the opportunities to peer at these behaviors), both wild and zoo-housed immature orangutans peered most in feeding, nest building, and social contexts (Figure 7B; see also^14^). Consequently, their relative peering frequencies in the different contexts did not differ significantly, with the exception of the feeding context. These results suggest that immatures at both settings selectively peer at behaviors that are either rare (i.e., they have limited social opportunities to peer at them) or more learning-intense, given their ecological and social environment.

### Conclusion

Our finding that wild and zoo-housed orangutans show similar age-dependent peering trajectories, despite vastly different social and ecological environments, suggests that immature orangutans' age-dependent tendency to peer is not affected by socio-ecological conditions and thus likely to some extent hard-wired. In other words, the relative tendency to attend to social information seems to develop largely independent of experience and prevailing conditions. However, the pronounced differences we found in peering rates, target choice, and contexts between wild and zoo-housed orangutans show that the frequency and the more fine-grained patterns of peering behavior are likely influenced by social and ecological factors, such as social opportunities to peer and the necessity to peer in learning-intense contexts. To what extent these factors act on the immediate versus the developmental level remains to be investigated, but the fact that zoo-housed orangutans show higher peering rates even after controlling for immediate differences in social opportunities (i.e., number of association partners that are currently in peering range) suggests that at least some of these effects are developmental. Our results demonstrate how social opportunities to learn from other individuals and the need to acquire learning-intense information were important preconditions for the evolution of cultures and thus may have also been a key factor in the evolution of human cognition.^20^

This study also highlights the general value of comparative studies of captive and wild populations of the same species. Our results show that zoo-housed individuals differ from wild individuals in their peering behavior, suggesting substantial plasticity in the expression of the behavior. The ability to adjust social information attendance in response to changing social and ecological conditions probably maximizes learning outcomes. This flexibility likely played a key role in the evolution of great ape culture, including human culture.^5^^,^^16^ However, our results also suggest that a species’ potential for attending to social information (and thus presumably also its social learning potential) can far exceed its naturally observed levels. Our findings therefore highlight that possible behavioral differences between zoo-housed and wild individuals need to be considered when seeking evolutionary interpretations for results from captivity. All in all, our study exemplifies that comparing the same species under a wide range of social and ecological conditions can provide a more complete picture of a behavior and shed light on the underlying mechanisms that regulate its expression.^38^

### Limitations of the study

Data on wild Sumatran orangutans were collected over many continuous years, resulting in a large dataset that includes many individuals at different developmental stages. Data collection in the zoos spanned only 26 months, resulting in a dataset with fewer age-individual data points. This sampling bias limited our analyses in some respects. For example, we could not test for sex-specific patterns in zoo-housed orangutans. Wild immature orangutans show sex-specific biases in their peering target selection with increasing age, likely due to their different dispersal backgrounds and thus the need to acquire different relevant ecological knowledge.^59^ So far, it remains unclear whether these peering biases are the result of intrinsic or developmental differences between the sexes. With more data from zoos on individuals of different sexes and at similar developmental stages, this question could be explored. Additionally, after arriving in a new area, wild migrating orangutan males selectively peer at local residents, suggesting that they utilize new learning opportunities.^40^ Selective attendance to local residents may also be observed and studied in relocated zoo-housed orangutans.

Although we found no statistically significant differences in peering rates between the zoos in the current dataset, visualization of the data suggests that this may have been due to the limited sample size per zoo (Figure S9). Differences in peering rates and other aspects of peering behavior among zoo-housed individuals may shed further light on the immediate and developmental factors affecting the behavior.

When looking at the effects of food item frequency on peering in zoo-housed orangutans, we assessed the frequency of a food item based on its occurrence in the adults’ diet on each follow day. However, this assessment does not take into account how often zoo-housed orangutans are exposed to the item on a day-to-day basis. In the wild, where fruit availability fluctuates and is measurable,^86^ this is easier to assess. Nonetheless, considering that we already see a clear trend in the zoo data (Figure 2B), it is likely that this trend would persist even if we accounted for overall food item frequency.

Lastly, we did not explore the potential of alternative functions of peering behavior in this study, such as peering as a means to solicit food from conspecifics (i.e., begging). In the wild, peering alone does not result in food transfer, and only 15.6% of food peering events are followed by begging behavior (i.e., begging gesture, attempt to take the food item).^14^ In the zoos, only 2% of all peering without begging lead to food transfer. Overall, peering in itself therefore appears to be an inefficient means of food acquisition and thus unlikely the main evolved function of the behavior.

## Resource availability

### Lead contact

Further information and requests for resources should be directed to and will be fulfilled by the lead contact, Paulina Kukofka (paulina.kukofka@uni-konstanz.de).

### Materials availability

No new materials were generated in this study.

### Data and code availability

• Data have been deposited at Mendeley and are publicly available as of the date of publication. The DOI is listed in the key resources table.
• All original code have been deposited at Mendeley and will be made publicly available as of the date of publication. The DOI is listed in the key resources table.
• Any additional information required to reanalyze the data reported in this paper is available from the lead contact upon request.

## Acknowledgments

We thank all researchers and students who conducted projects on peering at the Suaq Balimbing research site, and whose data were essential for this study, namely Natasha Bartolotta, Helvi Musdarlia, Anais van Cauwenberghe, Sonja Falkner, Luz Carvajal, Ellen Meulmann, Sofia Forss, Olivia Wassmer, Belinda Kunz, Beatrice Ehmann, Andrea Permana, Natialie Oliver-Caldwell, and Julia Mörchen. We acknowledge all students, volunteers, and local field assistants involved in the collection of standard behavioral data for the long-term database of Suaq Balimbing. We are thankful to the technicians and students who collected standard behavioral data in the zoos, especially to Ivan Lenzi, Francois Lamarque, Sree Subha Ramaswamy, Glenn Honstetter, Shubhangi Kansal, Nele Käter, and Mulati Mikeliban. Further, we gratefully acknowledge the curators, staff, and helpers at the zoo for their support in conducting our data collection: Adrian Baumeyer (Zoo Basel), Dr. Claudia Rudolf von Rohr (Zoo Zurich), Dr. Leyla Davis (Zoo Zurich), Dr. Wolfgang Ludwig (Zoo Dresden), and Roman Richter (Zoo Dresden). We thank our colleagues at the Max Planck Institute for Evolutionary Anthropology (MPI-EVA) in Leipzig and especially Hanna Petschauer and Dr. Daniel Hanus. We gratefully acknowledge the Badan Riset dan Inovasi Nasional (BRIN), the Taman Nasional Gunung Leuser (TNGL), in particular Arif Saifudin, Zakir, and Samsul Amar, the Universitas Nasional (UNAS), especially dean Dr. Fachruddin M. Mangunjaya, as well as the Yayasan Ekosistem Lestar (YEL) and its Sumatran Orangutan Conservation Survival Program (SOCP) for their permission and support for this research.

This study was funded through the Max Planck Institute of Animal Behavior (MPI-AB), the SUAQ Foundation, the 10.13039/501100006447University of Zurich, the Stiftung Mensch und Tier Freiburg i.Br., the A.H. Schultz Foundation, the 10.13039/100005966Leakey Foundation (Primate Research Fund), and the 10.13039/501100001663Volkswagen Stiftung (Freigeist Fellowship awarded to CS). SEA was supported by the Alexander von Humboldt Professorship endowed by the Federal Ministry of Education and Research awarded to Prof. Dr. Margaret Crofoot.

## Author contributions

Design and development of models, C.S., P.K., R.Y., and S.E.A.; analyzing models, C.S., P.K., and S.E.A.; design and conceptualizing research project, C.S. and P.K.; data collection and preparation, C.S., J.A.K., L.N., P.K., R.Y., and T.R.; writing manuscript, P.K.; review of the manuscript, all authors; supported field work, C.S., D.B.H., F.B., R.Y., and T.R.; supervision, C.S.

## Declaration of interests

The authors declare that the research was conducted in the absence of any commercial or financial relationships that could be construed as a potential conflict of interest.

## STAR★Methods

### Key resources table

**Table:** 

REAGENT or RESOURCE	SOURCE	IDENTIFIER
**Deposited data**		

Repository data	This paper	Kukofka, Paulina (2024), “Data_Kukofka et al. 2024”, Mendeley Data, V1, https://doi.org/10.17632/9g2w2fbjb9.1

**Experimental models: Organisms/strains**		

*Pongo abelii*	Wild Sumatran orangutans: Suaq Balimbing research station, 03°02′N; 97°25′E, Gunung Leuser National Park, South Aceh, Sumatra, Indonesia Zoo-housed orangutans: Zoo Zürich, Zürichbergstrasse 221, 8044 Zürich, Switzerland; Zoo Basel, Binningerstrasse 40, 4054 Basel, Switzerland; Zoo Dresden, Tiergartenstraβe 1, 01219 Dresden, Germany; Zoo Leipzig, Pfaffendorfer Str. 29, 04105 Leipzig, Germany	www.suaq.org www.zoo.ch www.zoobasel.ch www.zoo-dresden.de www.zoo-leipzig.de

**Software and algorithms**		

R 4.4.0	R Core Team. R: A Language and Environment for Statistical Computing. R Foundation for Statistical Computing (2024).^87^	www.r-project.org; RRID:SCR_001905

### Experimental model and study participant details

Data from the wild population of Sumatran orangutans (*Pongo abelii*) were collected over 12 years (October 2008 to January 2020) at the Suaq Balimbing monitoring station, located in the Gunung Leuser National Park in South Aceh, Indonesia (3°42′N, 97°26′E). Some of these data have been reported in previous studies (e.g.,^14^^,^^41^^,^^59^^,^^60^); however, with ongoing data collection, the dataset used for the current study is larger than the ones used for previous studies. Data on zoo-housed Sumatran orangutans were collected in zoos in Switzerland (Zoo Zürich and Zoo Basel) and Germany (Zoo Leipzig and Zoo Dresden) over two years (May 2021 to July 2023).

We included observations on 65 orangutans (40 from the wild, 25 from the zoos, Table S3). Thirty-five of the individuals were immatures at the time of data collection, all from known mothers, 22 from the wild (10 females, 12 males) and 13 housed in zoos (9 females, 4 males). The immatures’ age ranged from 0.5 to 15.9 years (mean = 5.8 years) in the wild and from 0.6 to 14.0 years (mean = 5.5 years) in the zoos. As the exact dates of birth for most of the individuals in the wild are unknown, we worked with estimates calculated by experienced observers upon first encounter of the animals. For wild adult females, ages are estimated based on their known number of offspring which is inferred based on observed dependent offspring and genetically confirmed offspring. For adult males with unknown birth dates, we assigned standardized birth dates: for unflanged males twenty years, and for flanged males thirty years, before the date on which they were first encountered in the study area.^88^ For orangutans born in the zoos, exact birth dates are known. Orangutans in zoo environments may differ in the time they reach developmental milestones such as age at first reproduction, but reliable data investigating developmental differences between zoo-housed and wild populations are missing to date. To simplify later analysis, we defined the age limits of each age class based on insights obtained from wild Sumatran orangutans: throughout this study “immatures” refers to all individuals below the age of sixteen years. Individuals below the age of eight years were classified as “dependent immatures” (based on the fact that at Suaq the average weaning age is 8.1 years, Schuppli unpublished data), and immatures between the age of 8.1 and 16 years as “independent immatures”.^46^^,^^89^

Group sizes in the zoo populations ranged from three (Basel and Dresden) to seven (Leipzig), and ten (Zurich) individuals at the time of data collection. Housing conditions were comparable between the zoos: all populations were regularly provisioned with food and with materials such as cardboard, wood wool, and blankets. They also had enrichment options such as puzzle boxes or tubes and access to an outdoor enclosure during the warmer seasons. We rarely observed aggression between individuals.

### Ethics approval and consent to participate

This study on wild and zoo-housed orangutans was strictly observational and non-invasive, and there was no interaction with our study animals. The research protocols for the observations at Suaq Balimbing were approved by the Indonesian State Ministry for Research, Technology and Higher Education (RISTEK; Research Permit No.: 152/SIP/FRP/SM/V/2012 and following) and complied with the legal requirements of Indonesia. At the zoos, the orangutans were observed from the visitor areas (or in the case of Leipzig Zoo, an observation tower). The observations did not interfere with the daily routine of the orangutans. The study complies with the Weatherall report titled ‘The use of non-human primates in research’,^90^ the EAZA Minimum Standards for the Accommodation and Care of Animals in Zoos and Aquaria,^91^ the WAZA Ethical Guidelines for the Conduct of Research on Animals by Zoos and Aquariums,^92^ and the ASAB/ABS’s Guidelines for the Treatment of Animals in Behavioural Research and Teaching.^93^ IAUCUC approval was not necessary to conduct this research. A joint ethical committee of the Max Planck Institute for Evolutionary Anthropology and Leipzig Zoo approved this study.

### Method details

#### Data collection

All data collected for this study were obtained through behavioral observations, following an established protocol for orangutan behavioral data collection (see https://www.ab.mpg.de/571325/standarddatacollectionrules_suaq_detailed_jan204.pdf). This study used two types of data: I) instantaneous scan sampling at 2-min intervals of the individual’s activity, its visibility, and its distance to other orangutans within a 50-meter radius (association partners), and II) all-occurrence sampling of peering behavior. Both data types were collected simultaneously during focal follows.

Peering is defined as “directly looking at the actions of another individual sustained over at least 5s, and at a close enough range that enables the peering individual to observe the details of the action” (see^14^). At every peering event, details such as the peering target and the activity were recorded. All observers (*n* = 17) whose data on wild orangutans were included in this study passed an interobserver reliability test of more than 85% accordance in simultaneous observations of the same individual. All observers (*n* = 18) in the zoos underwent training before collecting data and were closely supervised during follows to ensure accurate data entry. Overall, we included 8750 h and 1190 h of behavioral data on wild and zoo-housed orangutans, respectively, which resulted in 1915 observed peering events in the wild and 1186 peering events in the zoos.

### Quantification and statistical analysis

The full dataset included data from 1061 follow days of which 567 were from immature orangutans: 466 days from the wild data and 101 days from the zoo data (17 from Basel, 12 from Dresden, 25 from Leipzig, and 47 from Zurich). The dataset used for this study can be found at Mendeley Data: https://doi.org/10.17632/9g2w2fbjb9.1. The average observation duration on a follow day from immatures in the wild was 9.7 h (ranging from 0.3 to 13.6 h) and 8.2 h (ranging from 3.6 to 10.4 h) in the zoos, with 76% of the wild data, and 89% of the zoo data being full day follows (i.e., nest-to-nest in the wild, from opening until closing-time at the zoos). We used the all-occurrence data on peering behavior to analyze peering frequencies, peering target selection, and peering context selection, and the 2-min scan data to calculate overall visible observation duration, as well as time spent in peering range (0 to 2m) to association partners. To account for variations in the visibility of a focal individual during a follow, we excluded scans where the focal was out of the observer’s sight. In the wild, visibility has only been collected since 2014 but was positively correlated with age of the individual (LME, Estimate (Age 0–10) = 0.038, *p* < 0.001, Figure S8A and Table S4). For data collected before 2014, we used predicted visible observation duration values derived from the model estimates (Table S4).

All plots and all statistical analyses were performed using R version 4.4.0.^87^ All models included age (which we z-transformed) as a predictor to assess the effects of age on peering behavior. Visualization of the raw data showed that age had a non-linear effect on most of our response variables. To account for this non-linearity and ensure the best fit for our data, we ran Generalized Additive Mixed Models (GAMM) using the gam function from the mgcv package.^94^ We formulated all models which had counts as the response variable with a negative-binomial error distribution to avoid zero inflation issues caused by the large number of zeros in our datasets (as a result of follow days where no peering occurred, thus causing “true” zeros). For models with proportions as the response variable, we used a beta distribution.

Since we tested for differences in peering between the wild and the zoo, setting was included as a predictor in all models (with the exemption of models 1, and S1), as well as an interaction between age and setting, to test if age patterns in peering develop differently between settings. We modeled a smooth of age, and a tensor interaction between age and setting, to account for the non-linearity of these terms. We included individual ID as a random smooth in all models to avoid pseudo replication issues as individuals appeared multiple times in the datasets. To account for potential variation between different observers, we also included observer ID as a random smooth. Statistical significance was assessed at the 5% level for all analyses. We used the difference_smooths function of the gratia package^95^ to visually assess statistically significant differences between the age splines of each setting.

Aside from our main analyses, we tested whether individuals from different zoos significantly differed in their peering rates. Therefore, we fitted a GAMM model including age and site (i.e., Suaq, Zoo Basel, Zoo Dresden, Zoo Leipzig, Zoo Zürich), as fixed factors and individual ID and observer ID as random smooths (Figure S9 and Table S5). Visualization of the data suggests that individuals in zoos with smaller group sizes (Zoo Basel and Zoo Dresden) peered less frequently (Figure S9). However, post-hoc analysis showed that there were no statistically significant differences in peering rates between the different zoos (Table S6). Consequently, we did not distinguish between them in our main analyses.

#### Peering as a means for social learning

To assess whether peering is used as a means for social learning in dependent immature zoo-housed orangutans, we tested the effects of a food item’s processing intensity and frequency on peering behavior. We classified food items into difficult and easy-to-process food items. Difficult food items require multiple processing steps before ingestion, such as skilled manipulations to extract edible parts (e.g., peeling, breaking off, insertion of a tool). Easy food items can be ingested directly without prior processing (i.e., pick and eat) (see^14^^,^^96^). We counted peering events at easy and difficult food items per individual per day. Using the 2-min scan data we calculated the time adult association partners spent feeding on easy and difficult food items on that specific day. We then calculated peering rates controlled for opportunities to peer for each item processing class by dividing the number of peering events by the time adult association partners had spent feeding on it. To obtain a measure of the overall frequency of a food item, we divided the time adult association partners spent feeding on each item by the total visible observation duration. To calculate peering rates controlled for opportunities to peer at the different food items, we divided the number of peering events directed at each food item by the time that food item was consumed by association partners on that specific day. We analyzed a total of 238 peering events from seven dependent immatures and 25 follow days. In the model, we set the dependent immatures’ peering events (for easy and difficult food items, respectively) as the response variable, and the time adult association partners spent feeding on easy or difficult food items as the offset term (Model 1). The age of the immatures, processing intensity, and frequency were included as predictors.

#### Ontogeny of peering

To look at the development of peering over age we used the full dataset, i.e., of all immature and adult individuals (see above). We calculated daily peering rates by dividing total counts of peering events by the visible observation duration on each day. The model included peering counts as the response variable and the visible observation duration as the offset term (Model 2a). As most peering occurs during immaturity, we focused the more detailed analyses on this age class. To account for different social opportunities to peer, we calculated peering rates that were controlled for the availability of association partners by dividing the number of peering events by the time immatures spent in peering range to at least one association partner. We included 2993 peering events from 538 individual follows in this analysis. The model included peering counts as the response variable and the time immatures spent in peering range as the offset term (Model 2b).

#### Development of peering target selection

To investigate the development of immatures’ peering target selection, we counted and grouped peering events directed at either the mother or non-mother individuals. We used counts of peering events with the mother and with non-mother targets as response variables, respectively, and included the visible observation duration as the offset term in the models (Models 3a, and 3b). To account for varying opportunities to peer at the two classes of peering targets, we calculated peering rates as the number of peering events divided by the time the observed individual spent in peering range to the respective class of target (i.e., we controlled for opportunities to peer). To obtain proportions of peering, we then divided peering rates by the sum of all peering rates obtained of each class of target. The model included the proportion of immatures’ peering at non-mother targets as the response variable (Model 3c). Further, to investigate the development of peering target selection in terms of peering targets ages’ relative to the age of the peerer, we divided individuals into four age intervals: 0–4 years, 4–8 years, 8–16 years, and 16+ years. Individuals within the same age interval were considered as peers. To account for varying opportunities to peer at the different relative age classes, we calculated proportions of peering as described above. For this analysis we included data from the wild, data from Zoo Zürich and from Zoo Leipzig. Target selection in Zoo Basel and Zoo Dresden was limited to the immature’s parents due to the group composition. This resulted in 238 follows from the wild and 70 follows from the zoos in which peering occurred. In the models, we used the proportions of peering at older, same-aged (‘peers’), and younger individuals as response variables, respectively (Models 3d-f).

#### Peering context selection

We divided peering contexts into five categories: feeding, nesting, social, exploratory, and other (i.e., all non-social activities, such as resting, moving, and grooming of oneself) behavior. We calculated the overall proportions of peering in each context by dividing the number of peering events in each context by the total number of peering events for each individual per focal follow day. We controlled for differing opportunities to peer in each context by first calculating peering rates by dividing the number of peering events in each context by the time adult association partners spent performing the behavior per day. We analyzed 2797 peering events by 24 dependent immatures (16 from the wild, and 8 from the zoos) collected on 291 individual follows. We first fitted models using the proportions of peering in the respective contexts as the response variable (Models 4a-e). We then compared the actual rates of peering by setting the peering counts in the respective contexts as the response variable, with the time association partners spent engaging in the behaviors as the offset term (Models 4f-j).
