# Supplementary material for: The development of social attention in orangutans: Comparing peering behavior in wild and zoo-housed individuals
Source: iScience. 2024 Dec 6;28(1):111542. doi: 10.1016/j.isci.2024.111542 (PMC11732129; doi:10.1016/j.isci.2024.111542)
Supplement: Document S1. Figures S1–S9 and Tables S1–S6 [file mmc1.pdf]

iScience, Volume *28*

Supplemental information

The development of social attention in orangutans: Comparing peering behavior in wild and zoo-housed individuals

Paulina Kukofka, Richard Young, Julia A. Kunz, Lara Nellissen, Shauhin E. Alavi, Tri Rahmaeti, Fitriah Basalamah, Daniel B.M. Haun, and Caroline Schuppli

SUPPLEMENTAL INFORMATION

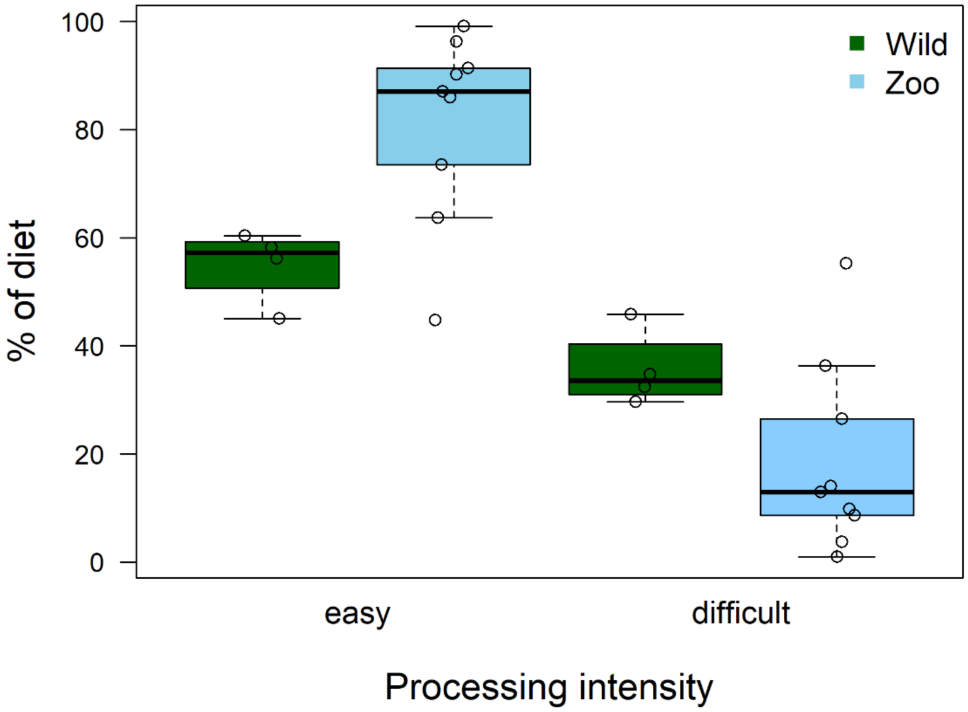

Figure S1. Comparison of processing intensity of the two settings. Related to Introduction and Discussion. Percentage of easy and difficult food items in the diet of adult wild and zoo-housed orangutans. Based on data of 4 adult females in at Suaq, and of 9 adult individuals (2 males and 7 females) in the zoos. Percentages were calculated based on time spent feeding on the different item types.

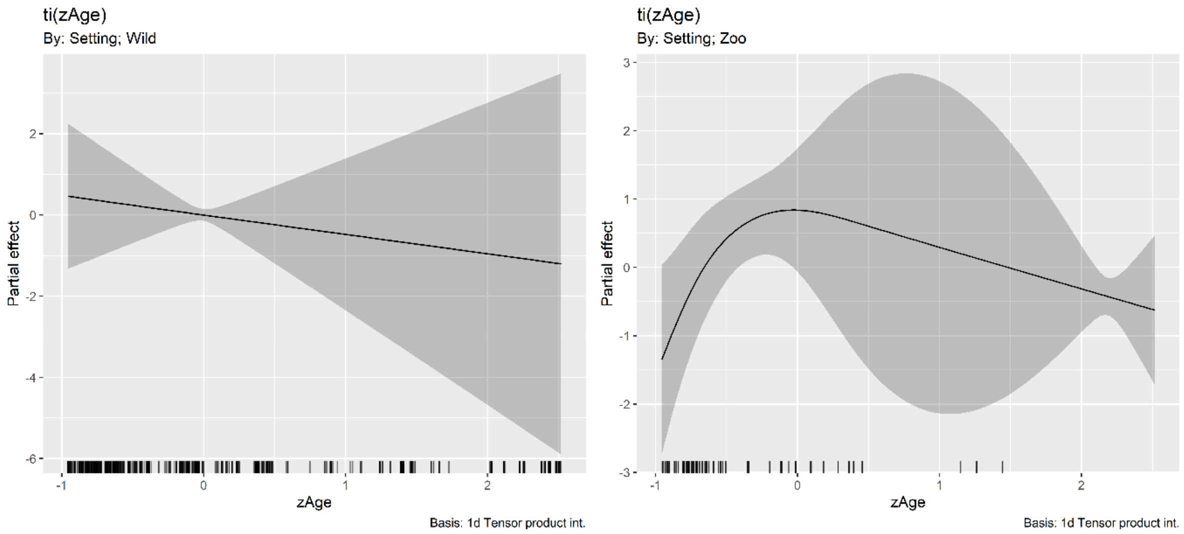

Figure S2. Smoothing splines for age for each setting. Related to Figure 3. Smoothing splines for the tensor interaction between age and the two settings in Model 2a.

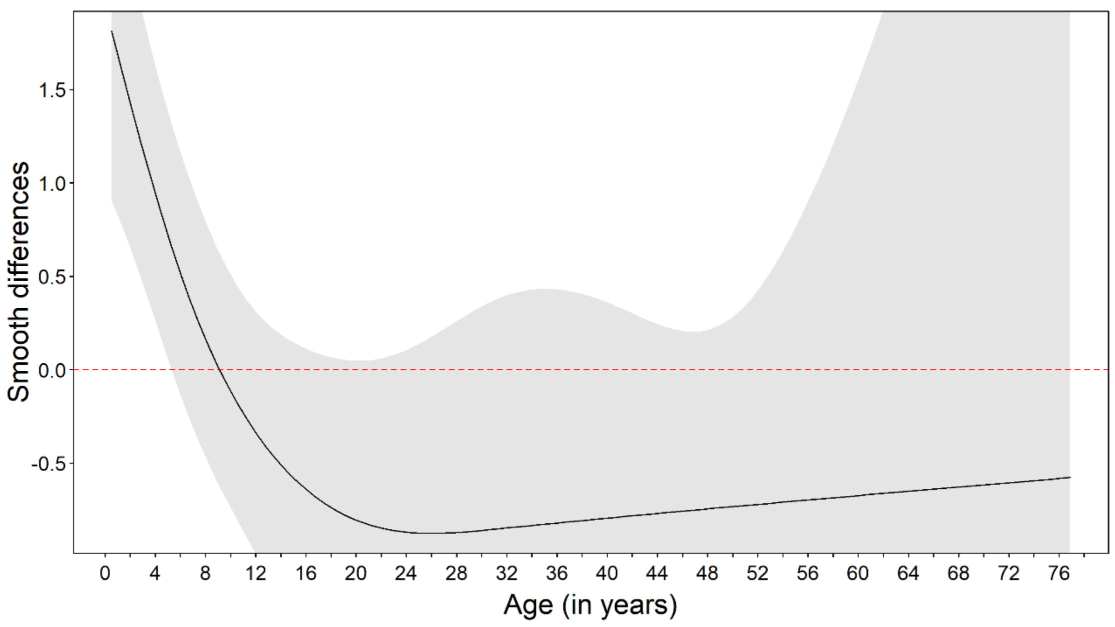

Figure S3. Differences in age trends in peering frequency. Related to Figure 3. Differences between the smoothing splines of the two settings in Model 2a for peering in the wild and in the zoos over age. Grey areas are 95 % confidence intervals. Areas of statistical significance at the 5 % level are assesses by whether or where y = 0 (red dotted line) crosses the confidence interval.

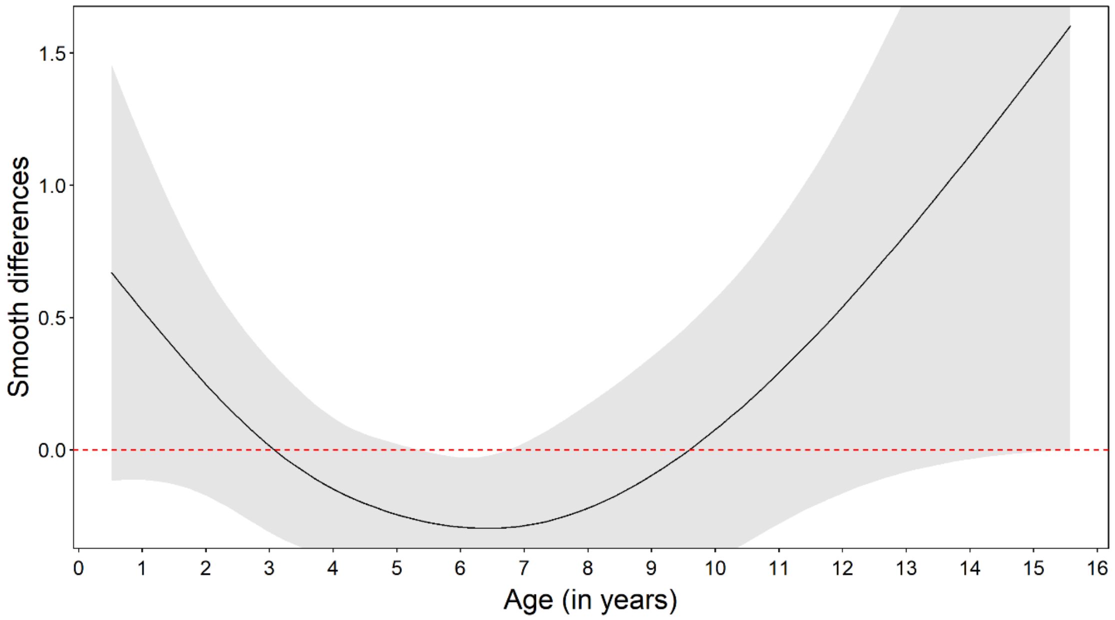

Figure S4. Differences in age trends in peering frequency controlled for the availability of association partners. Related to Figure 4. Differences between the smoothing splines of the two settings in Model 2b for peering in peering range in the wild and the zoos over age. Grey areas are 95 % confidence intervals. Areas of statistical significance at the 5 % level are assesses by whether or where y = 0 (red dotted line) crosses the confidence interval.

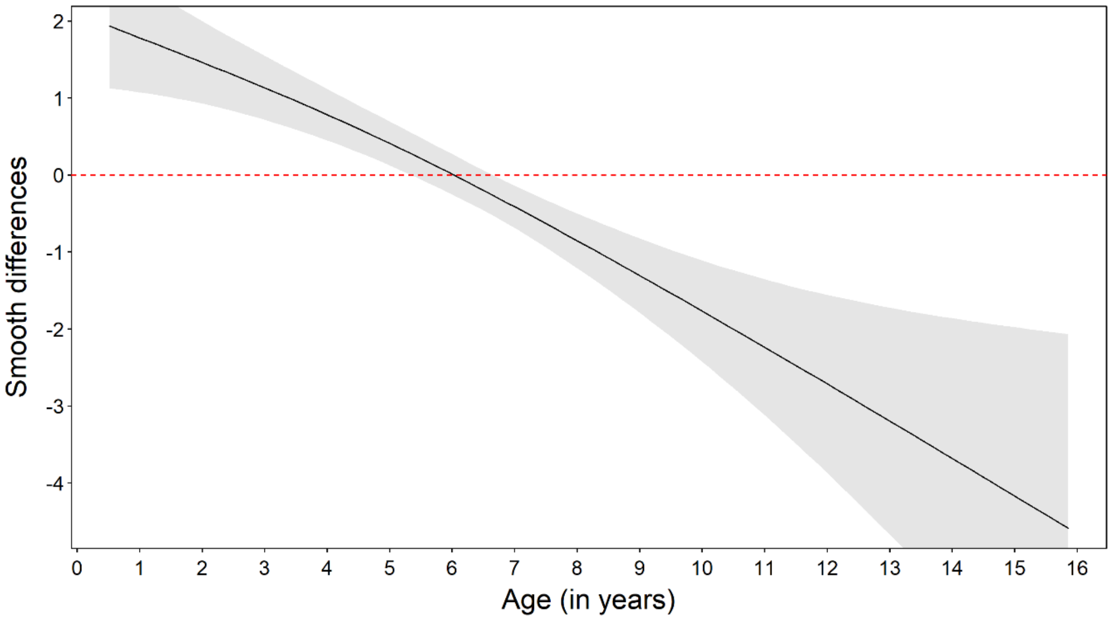

Figure S5. Differences in age trends in peering frequency at the mother. Related to Figure 5a. Differences between the smoothing splines of the two settings in Model 3a for peering directed at the mother in the wild and the zoos over age. Grey areas are 95 % confidence intervals. Areas of statistical significance at the 5 % level are assesses by whether or where y = 0 (red dotted line) crosses the confidence interval.

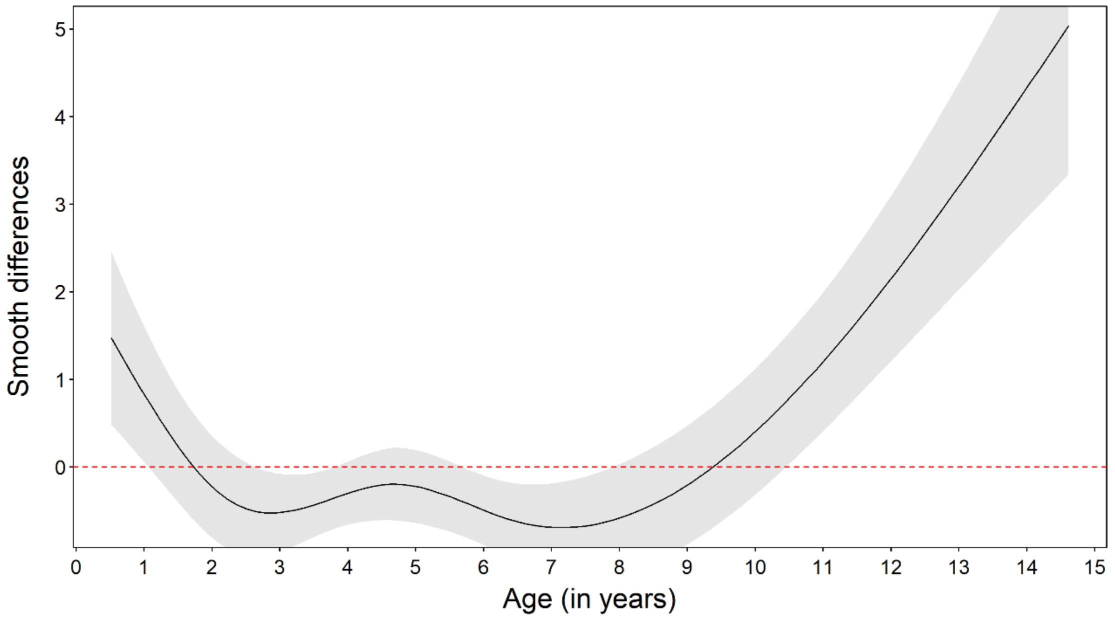

Figure S6. Differences in age trends for peering frequencies at non-mother targets controlled for opportunities to peer. Related to Figure 6a. Differences between the smoothing splines of the two settings in Model 3c for peering proportions directed at non-mother individuals in the wild and the zoos over age. Grey areas are 95 % confidence intervals. Areas of statistical significance at the 5 % level are assesses by whether or where y = 0 (red dotted line) crosses the confidence interval.

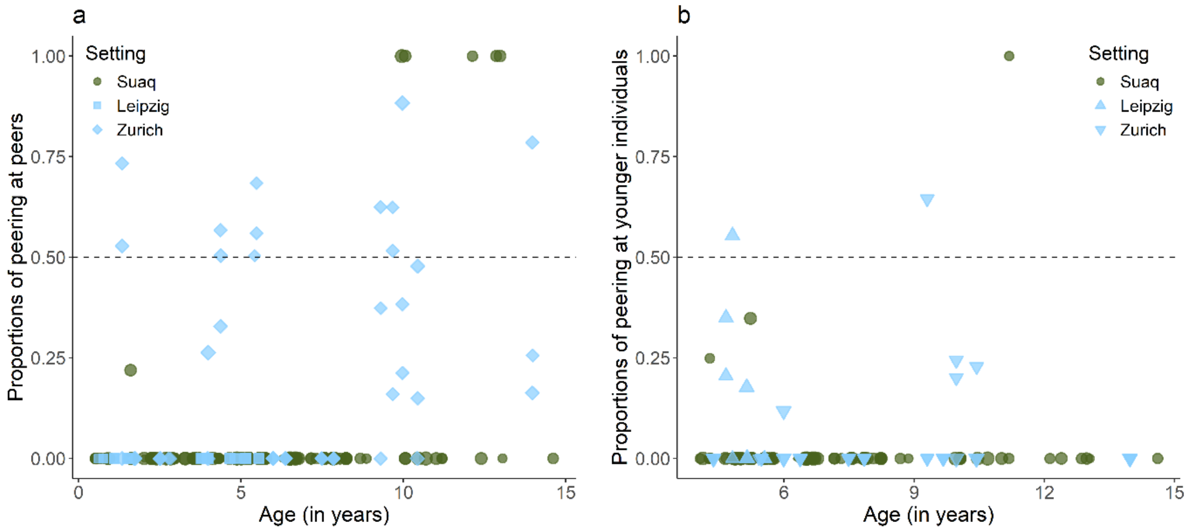

Figure S7. Development of peering frequencies at peering targets of different relative ages controlled for opportunities to peer. Related to Figure 6b. Proportions of peering by wild and zoo-housed immature orangutans directed at (a) peers, and (b) younger individuals controlled for the time the peering individual spent in close proximity to the peering targets. Each point represents one focal follow day. Symbol sizes correspond to the log values of the total number of observed peering events.

Table S1. Peering target selection: summary of models 3e and 3f. GAMMs with setting (Wild / Zoo), age (z-transformed), as well as an interaction between age and setting as fixed effects, and individual and observer ID as random effects. Listed are estimates, standard errors, p values, sample size (n), adjusted r^2, and deviance explained (DE). P values with significance at the 5 % level are indicated in bold font.

Model	Response variable	Predictors	Type	Estimate	Standard Error	p-value
3e	Proportions	Parametric terms:				
	of peering	(Intercept)	Intercept	-2.663	0.285	**< 0.001**
n = 308	at peers	Setting (zoo)	Fixed effect	0.915	0.501	0.068
$r^2_{adj.}$ =	(immatures)	Smooth terms:				
0.59		Z-Age	Fixed effect			**< 0.001**
DE = 71.4		Z-Age:Wild	Interaction			1.000
%		Z-Age:Zoo	Interaction			0.112
		Individual ID	Random effect			**< 0.001**
		Observer ID	Random effect			**< 0.001**
3f	Proportions	Parametric terms:				
	of peering	(Intercept)	Intercept	-2.614	0.122	**< 0.001**
n = 167	at younger	Z-Age	Fixed effect	0.027	0.046	0.557
$r^2_{adj.}$ =	individuals	Setting (zoo)	Fixed effect	0.361	0.156	**0.021**
0.12	(immatures)	Z-Age:Zoo	Interaction	-0.014	0.091	0.880
DE = 79 %		Smooth terms:				
		Individual ID	Random effect			0.455
		Observer ID	Random effect			**< 0.001**

Table S2. Context selection: summaries for models 4a-j. Related to Figure 7. GAMMs with setting (Wild/Zoo), age (z-transformed), as well as an interaction between age and setting as fixed effects, and individual and observer ID as random effects. Models 4f-j included the time association partners spent engaging in the behaviour as the offset term. Listed are estimates, standard errors, p values, sample size (n), adjusted r^2, and deviance explained (DE). P values with significance at the 5 % level and corrected for multiple testing due to the dependence in the response variable are indicated in bold font.

Model	Response variable	Predictors	Type	Estimate	Standard Error	p-value
4a	Proportion	Parametric terms:				
	of peering	(Intercept)	Intercept	1.268	0.170	**< 0.001**
n = 246	at feeding	Setting (zoo)	Fixed effect	-1.253	0.270	**< 0.001**
$r^2_{adj.}$ =	behaviour	Smooth terms:				
0.33	(dependent	Z-Age	Fixed effect			0.110
DE =	immatures)	Z-Age:Wild	Interaction			0.323
28.6 %		Z-Age:Zoo	Interaction			0.973
		Individual ID	Random effect			**0.003**
		Observer ID	Random effect			0.756
4b	Proportion	Parametric terms:				
	of peering	(Intercept)	Intercept	-1.223	0.094	**< 0.001**
n = 246	at nesting	Z-Age	Fixed effect	-0.031	0.138	0.821
$r^2_{adj.}$ =	behaviour	Setting (zoo)	Fixed effect	-0.561	0.164	**< 0.001**
0.10	(dependent	Smooth terms:				
DE =	immatures)	Z-Age:Wild	Interaction			0.240
42.8 %		Z-Age:Zoo	Interaction			0.999
		Individual ID	Random effect			0.498
		Observer ID	Random effect			0.876
4c	Proportion	Parametric terms:				
	of peering	(Intercept)	Intercept	-3.669	0.078	**< 0.001**
n = 246	at social	Z-Age	Fixed effect	-0.022	0.078	0.772
$r^2_{adj.}$ =	behaviour	Setting (zoo)	Fixed effect	0.499	0.144	**< 0.001**
0.04	(dependent	Smooth terms:				
DE = 9.3	immatures)	Z-Age:Wild	Interaction			0.998
%		Z-Age:Zoo	Interaction			0.911

		Individual ID	Random effect			0.887
		Observer ID	Random effect			0.999
4d	Proportion	<u>Parametric terms:</u>				
	of peering	(Intercept)	Intercept	-2.472	0.083	**< 0.001**
n = 246	at	Z-Age	Fixed effect	0.011	0.153	0.941
$r^2_{adj.}$ =	exploratory	Setting (zoo)	Fixed effect	1.706	0.170	**< 0.001**
0.48	behaviour	<u>Smooth terms:</u>				
DE =	(dependent	Z-Age:Wild	Interaction			1.000
41.7 %	immatures)	Z-Age:Zoo	Interaction			**0.031**
		Individual ID	Random effect			0.740
		Observer ID	Random effect			0.913
4e	Proportion	<u>Parametric terms:</u>				
	of peering	(Intercept)	Intercept	-3.617	0.078	**< 0.001**
n = 246	at other	Z-Age	Fixed effect	-0.107	0.128	0.406
$r^2_{adj.}$ =	(non-	Setting (zoo)	Fixed effect	0.432	0.144	**< 0.001**
0.03	social)	<u>Smooth terms:</u>				
DE =	behaviour	Z-Age:Wild	Interaction			0.556
8.52 %	(dependent	Z-Age:Zoo	Interaction			0.220
	immatures)	Individual ID	Random effect			0.824
		Observer ID	Random effect			0.887
4f	Peering	<u>Parametric terms:</u>				
	counts at	(Intercept)	Intercept	-1.281	0.271	**< 0.001**
n = 363	feeding	Setting (zoo)	Fixed effect	1.634	0.290	**< 0.001**
$r^2_{adj.}$ =	behaviour	<u>Smooth terms:</u>				
0.50	(dependent	Z-Age	Fixed effect			**0.003**
DE =	immatures)	Z-Age:Wild	Interaction			**< 0.001**
63.6 %		Z-Age:Zoo	Interaction			0.980
		Individual ID	Random effect			**< 0.001**
		Observer ID	Random effect			**< 0.001**
4g	Peering	<u>Parametric terms:</u>				
	counts at	(Intercept)	Intercept	0.108	0.314	0.730
n = 309	nesting	Z-Age	Fixed effect	-0.309	0.738	0.676
$r^2_{adj.}$ =		Setting (zoo)	Fixed effect	-0.231	0.568	0.685
0.30		<u>Smooth terms:</u>				

DE =	behaviour	Z-Age:Wild	Interaction			**0.032**
35.7 %	(dependent	Z-Age:Zoo	Interaction			0.188
	immatures)	Individual ID	Random effect			0.113
		Observer ID	Random effect			**< 0.001**
4h	Peering	Parametric terms:				
	counts at	(Intercept)	Intercept	-1.823	0.520	**< 0.001**
n = 232	social	Z-Age	Fixed effect	-0.695	0.260	**0.007**
$r^2_{adj.}$ =	behaviour	Setting (zoo)	Fixed effect	0.713	0.585	0.223
0.20	(dependent	Smooth terms:				
DE =	immatures)	Z-Age:Wild	Interaction			0.156
24.5 %		Z-Age:Zoo	Interaction			**< 0.001**
		Individual ID	Random effect			0.224
		Observer ID	Random effect			0.093
4i	Peering	Parametric terms:				
	counts at	(Intercept)	Intercept	-1.176	2.161	0.586
n = 118	exploratory	Z-Age	Fixed effect	1.633	3.116	0.600
$r^2_{adj.}$ =	behaviour	Setting (zoo)	Fixed effect	1.029	1.711	0.549
0.45	(dependent	Smooth terms:				
DE = 18	immatures)	Z-Age:Wild	Interaction			0.586
%		Z-Age:Zoo	Interaction			0.710
		Individual ID	Random effect			**0.036**
		Observer ID	Random effect			0.244
4j	Peering	Parametric terms:				
	counts at	(Intercept)	Intercept	-5.484	0.737	**< 0.001**
n = 363	other (non-	Setting (zoo)	Fixed effect	2.597	0.988	**0.009**
$r^2_{adj.}$ =	social)	Smooth terms:				
0.52	behaviour	Z-Age	Fixed effect			**0.046**
DE =	(dependent	Z-Age:Wild	Interaction			0.339
73.2 %	immatures)	Z-Age:Zoo	Interaction			**0.036**
		Individual ID	Random effect			**0.015**
		Observer ID	Random effect			**0.004**

Table S3. Overview table of all individuals included in this study. Related to STAR Methods. The table shows the amount of data points per individual (N) in the full data sets, site of data collection, focal name, age classes observed, observation period, and observed age range.

N	Site	Focal	Age class	Sex	Observation period	Age range (y)
12	Suaq	Alice	Adult	Female	2010, 2017/18, 2020	50.5 – 59.5
5	Suaq	Amor	Immature	Male	2017/18	2.3 – 3.5
4	Suaq	Balu	Adult	Male	2017, 2019	24.7 – 26.8
7	Suaq	Caesar	Adult	Male	2018/19	21.6 – 23.1
12	Suaq	Chindy	Immature	Female	2008, 2010/11, 2017	5.8 – 14.6
61	Suaq	Cinnamon	Immature	Female	2013/14, 2016 – 2019	1.6 – 7.6
68	Suaq	Cissy	Adult	Female	2010/11, 2013/14, 2017 – 2019	45.8 – 54.3
4	Suaq	Derek	Adult	Male	2017 – 2019	23.6 – 25.3
2	Suaq	Dian	Adult	Male	2009, 2019	31.2 – 41.2
2	Suaq	Diddy	Immature	Male	2009, 2011	3.2 – 5.1
4	Suaq	Dodi	Adult	Female	2009, 2011, 2013	40.6 – 45.4
4	Suaq	Dru	Adult	Male	2018	24.3 – 24.4
68	Suaq	Eden	Immature	Female	2017 – 2020	2.2 – 5.2
92	Suaq	Ellie	Adult	Female	2009 – 2011, 2017 – 2020	17.9 – 20.9
74	Suaq	Frankie	Immature	Male	2013/14, 2016 – 2019	0.9 – 7.3
30	Suaq	Fredy	Immature	Male	2008 – 2011, 2013/14	3.5 – 8.7
99	Suaq	Friska	Adult	Female	2008 – 2011, 2013/14, 2016 – 2019	65.9 – 76.9
4	Suaq	Gura	Adult	Male	2017/18	29.5 – 30.5
7	Suaq	Islo	Adult	Male	2008, 2018/19	31.2 – 42.3
2	Suaq	Karma	Adult	Female	2008, 2011	13.5 – 15.6
30	Suaq	Lilly	Immature – Adult	Female	2009, 2011, 2013, 2017 – 2019	7.9 – 18.0
115	Suaq	Lisa	Adult	Female	2008/9, 2011, 2013/14, 2016 – 2019	21.3 – 32.3

104	Suaq	Lois	Immature	Male	2011, 2013/14, 2016 – 2019	0.5 – 9.3
9	Suaq	Luther	Immature	Male	2017 – 2019	1.2 – 3.0
4	Suaq	Marco	Adult	Male	2018	20.8
1	Suaq	Milo	Adult	Male	2009	20.3
1	Suaq	Otto	Adult	Male	2017	40.0
7	Suaq	Pepito	Immature	Male	2017 – 2019	4.5 – 6.9
4	Suaq	Pluto	Adult	Male	2017	34.4 – 34.6
12	Suaq	Raffi	Adult	Female	2014, 2017	41.2 – 44.6
11	Suaq	Rendang	Immature	Male	2014	0.7 – 0.9
1	Suaq	Ronaldo	Immature	Male	2011	5.0
4	Suaq	Sazu	Immature	Male	2014, 2017/18	7.6 – 11.9
8	Suaq	Shera	Immature	Female	2010/11	12.8 – 13.1
5	Suaq	Simba	Immature	Male	2014	0.9
9	Suaq	Tina	Immature	Female	2008, 2011	10.9 – 13.2
7	Suaq	Tornado	Immature	Male	2017 – 2019	2.8 – 4.7
10	Suaq	Trident	Immature	Female	2016 – 2019	10.4 – 13.2
1	Suaq	Yoyo	Adult	Male	2009	17.1
11	Suaq	Yulia	Immature	Female	2016, 2018	9.8 – 11.7
3	Basel Zoo	Budi	Adult	Male	2021	17.3
3	Zurich Zoo	Cahaya	Adult	Female	2021/22	19.1 – 20.2
8	Dresden Zoo	Daisy	Adult	Female	2021/22	30.2 – 31.6
12	Dresden Zoo	Dalai	Immature	Male	2021/2022	5.8 – 7.2
3	Dresden Zoo	Djaka	Adult	Female	2022	26.3
3	Zurich Zoo	Djarius	Adult	Male	2022	27.8
3	Dresden Zoo	Djudi	Adult	Female	2022	49.3
3	Zurich Zoo	Hadiah	Immature	Male	2021	14
5	Basel Zoo	Ketawa	Immature	Female	2021	8.5
10	Leipzig Zoo	Lursa	Immature	Female	2022/23	0.6 – 1.5
3	Basel Zoo	Maia	Adult	Female	2021	13.8
6	Zurich Zoo	Malou	Immature	Male	2021/22	9.7 – 10.4
6	Zurich Zoo	Mimpi	Immature	Female	2021/22	9.3 – 10.0

3	Basel Zoo	Ombak	Immature	Male	2022	5.3
5	Leipzig Zoo	Padana	Adult	Female	2021	23.5 – 23.7
6	Basel Zoo	Padma	Immature	Female	2021/23	3.1 – 4.6
12	Zurich Zoo	Pandai	Immature	Female	2021 - 2023	6.0 – 7.9
3	Leipzig Zoo	Raja	Adult	Female	2022	19.0
6	Basel Zoo	Revital	Adult	Female	2021	21.2 – 21.3
9	Zurich Zoo	Riang	Immature	Female	2021/22	4.0 – 5.5
15	Leipzig Zoo	Sari	Immature	Female	2021 – 2023	3.8 – 5.6
2	Zurich Zoo	Timor	Adult	Female	2022	46.8
3	Dresden Zoo	Toni	Adult	Male	2021	29.6
11	Zurich Zoo	Utu	Immature	Female	2021/22	1.3 – 2.8
3	Zurich Zoo	Xira	Adult	Female	2023	25.6

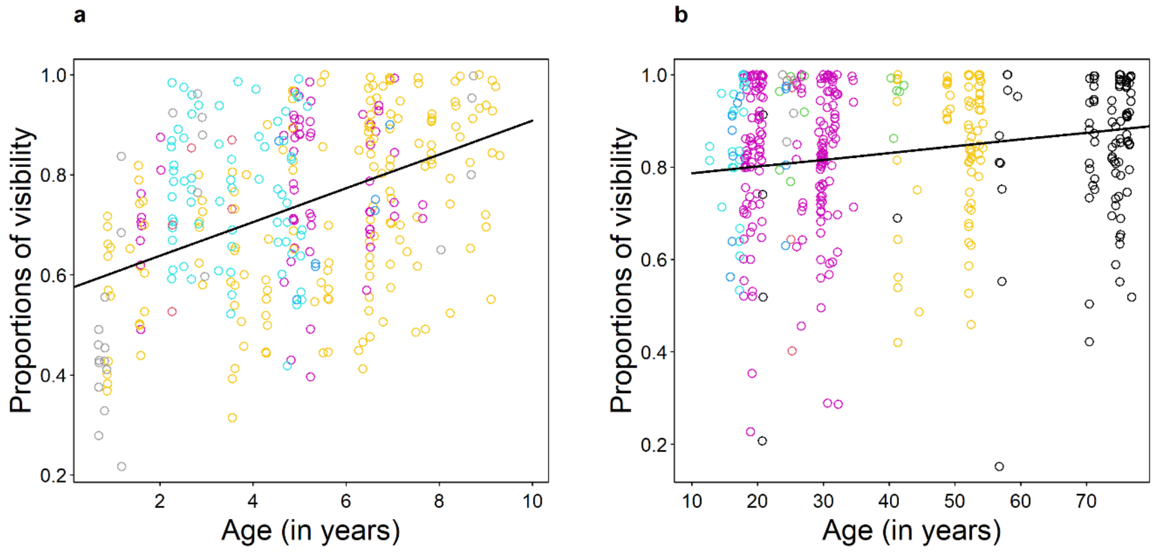

Figure S8. Proportion of focal visibility over age in the wild population. Related to STAR Methods. Proportion of visible observation time of each focal follow day in Suaq, Indonesia over age for (a) 0 to 10 years, and (b) 10+ years. Each point represents one focal follow day. Colours refer to different individuals. The lines represent mean model predictions (plotted using the *ggpredict* function of the ggeffects package [S1]).

Table S4. Model summary for model M1a+b. Related to STAR Methods. Linear Mixed-Effects Model for focal visibility in the wild data set. Analysis conducted using the *lmer* function of the lme4 package [S2]. The table shows estimates, standard errors, P values, sample size (N) and confidence intervals. P values with significance at the 5 % level are indicated in bold font (assessed with the lmerTest package [S3]). CI = confidence interval.

Model	Dependent variable	Effect	Effect type	Estimate	Std. Error	P value	95 % CI
M1a<br><br>n = 344	Proportion of visibility (0 – 10 years)	(Intercept)<br>Age<br>Individual ID	Intercept<br>Fixed effect<br>Random effect	0.571<br>0.038<br>-	0.030<br>0.004<br>-	**<0.001**<br>**<0.001**<br>-	0.512 – 0.629<br>0.025 – 0.042<br>-
M1b<br><br>n = 423	Proportion of visibility (10+ years)	(Intercept)<br>Age<br>Individual ID	Intercept<br>Fixed effect<br>Random effect	0.772<br>0.001<br>-	0.038<br>0.001<br>-	**<0.001**<br>0.186<br>-	0.697 – 0.846<br>-0.001 – 0.003

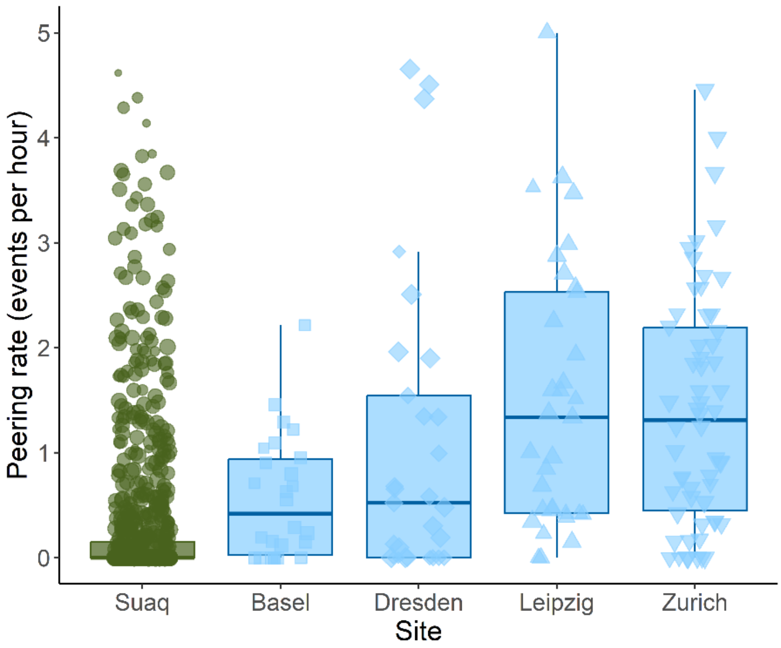

Figure S9. Peering frequency per site. Related to Limitations of the study and STAR Methods. Mean hourly peering rates across the five sites of data collection. The bold horizontal line indicates the median, the box the lower and upper quartile, the whiskers the lowest and greatest value, respectively, and circles are outliers. Each point represents one focal follow day.

Table S5. Development of peering frequency over age: summary for model 2c. Related to STAR Methods. GAMM with site (Suaq, Basel, Dresden, Leipzig, Zurich), age (z-transformed), as well as an interaction between age and site as fixed effects, and individual and observer ID as random effects. The model included visible observation duration as an offset term. Listed are estimates, standard errors, p values, sample size (n), adjusted r^2, and deviance explained (DE). P values with significance at the 5 % level are indicated in bold font.

Model	Response variable	Predictors	Type	Estimate	Standard Error	p-value
2c	Peering	Parametric terms:				
	counts	(Intercept)	Intercept	-3.308	0.239	**< 0.001**
n = 1061	total (all	Site (Zoo Basel)	Fixed effect	0.032	0.677	0.963
$r^2_{adj.}$ = 0.63	ages)	Site (Zoo Dresden)	Fixed effect	2.634	0.374	**< 0.001**
DE =		Site (Zoo Leipzig)	Fixed effect	3.463	5.243	0.509
73.7%		Site (Zoo Zürich)	Fixed effect	2.173	0.404	**< 0.001**
		Smooth terms:				
		Z-Age	Fixed effect			**< 0.001**
		Z-Age:Suaq	Interaction			**< 0.001**
		Z-Age:Zoo Basel	Interaction			0.998
		Z-Age:Zoo Dresden	Interaction			**0.014**
		Z-Age:Zoo Leipzig	Interaction			**0.020**
		Z-Age:Zoo Zurich	Interaction			0.065
		Individual ID	Random effect			**0.002**
		Observer ID	Random effect			**< 0.001**

Table S6. Comparison between peering frequency per sites. **Related to STAR Methods.** Post-hoc pairwise comparison of estimated marginal means of the data collection sites (Model A2), using the *emmeans* function from the emmeans package [S4]. The table shows contrasts, estimates, standard errors, t-ratio, and p values for each comparison. P values with significance at the 5 % level are indicated in bold font.

Contrast	Estimate	Standard Error	t-ratio	p-value
Suaq – Zoo Basel	-1.694	0.719	-2.356	0.129
Suaq – Zoo Dresden	-4.223	0.455	-9.284	**< 0.001**
Suaq – Zoo Leipzig	-4.085	0.554	-7.377	**< 0.001**
Suaq – Zoo Zürich	-3.835	0.473	-8.114	**< 0.001**
Zoo Basel – Zoo Dresden	-2.529	0.706	-3.582	0.003
Zoo Basel – Zoo Leipzig	-2.391	0.785	-3.046	0.020
Zoo Basel – Zoo Zürich	-2.142	0.719	-2.978	0.025
Zoo Dresden – Zoo Leipzig	0.138	0.553	0.250	1.000
Zoo Dresden – Zoo Zürich	0.388	0.445	0.871	0.908
Leipzig Zoo – Zoo Zürich	0.250	0.558	0.448	0.992

REFERENCES

[S1] D. Lüdecke, "ggeffects: Tidy Data Frames of Marginal Effects from Regression Models," *JOSS*, vol. 3, no. 26, p. 772, Jun. 2018, doi: 10.21105/joss.00772.

[S2] D. Bates, M. Mächler, B. Bolker, and S. Walker, "Fitting Linear Mixed-Effects Models Using lme4," *J. Stat. Soft.*, vol. 67, no. 1, pp. 1–48, Oct. 2015, doi: 10.18637/jss.v067.i01.

[S3] A. Kuznetsova, P. B. Brockhoff, and R. H. B. Christensen, "lmerTest Package: Tests in Linear Mixed Effects Models," *J. Stat. Soft.*, vol. 82, no. 13, pp. 1–26, Dec. 2017, doi: 10.18637/jss.v082.i13.

[S4] R. V. Lenth, *emmeans: Estimated Marginal Means, aka Least-Squares Means*. (2024). [Online]. Available: https://CRAN.R-project.org/package=emmeans
